# Restoring carboxypeptidase E rescues BDNF maturation and neurogenesis in aged brains

**DOI:** 10.1093/lifemedi/lnad015

**Published:** 2023-04-11

**Authors:** Hongmei Liu, Dongfang Jiang, Fuwen Yao, Tingting Li, Bo Zhou, Song Zhao, Keyan Yang, Haiping Feng, Jiaqi Shen, Jinglan Tang, Sijia Wang, Yu-Xin Zhang, Yun Wang, Qian Li, Yongliang Zhao, Caixia Guo, Tie-Shan Tang

**Affiliations:** State Key Laboratory of Membrane Biology, Institute of Zoology, Chinese Academy of Sciences, Beijing 100101, China; Institute for Stem cell and Regeneration, Chinese Academy of Sciences, Beijing 100101, China; Beijing Institute for Stem Cell and Regenerative Medicine, Beijing 100101, China; State Key Laboratory of Membrane Biology, Institute of Zoology, Chinese Academy of Sciences, Beijing 100101, China; University of Chinese Academy of Sciences, Chinese Academy of Sciences, Beijing 100101, China; Beijing Institute of Genomics, Chinese Academy of Sciences/China National Center for Bioinformation, Beijing 100101, China; University of Chinese Academy of Sciences, Chinese Academy of Sciences, Beijing 100101, China; State Key Laboratory of Membrane Biology, Institute of Zoology, Chinese Academy of Sciences, Beijing 100101, China; University of Chinese Academy of Sciences, Chinese Academy of Sciences, Beijing 100101, China; Beijing Institute of Genomics, Chinese Academy of Sciences/China National Center for Bioinformation, Beijing 100101, China; State Key Laboratory of Membrane Biology, Institute of Zoology, Chinese Academy of Sciences, Beijing 100101, China; University of Chinese Academy of Sciences, Chinese Academy of Sciences, Beijing 100101, China; State Key Laboratory of Membrane Biology, Institute of Zoology, Chinese Academy of Sciences, Beijing 100101, China; University of Chinese Academy of Sciences, Chinese Academy of Sciences, Beijing 100101, China; State Key Laboratory of Membrane Biology, Institute of Zoology, Chinese Academy of Sciences, Beijing 100101, China; University of Chinese Academy of Sciences, Chinese Academy of Sciences, Beijing 100101, China; State Key Laboratory of Membrane Biology, Institute of Zoology, Chinese Academy of Sciences, Beijing 100101, China; University of Chinese Academy of Sciences, Chinese Academy of Sciences, Beijing 100101, China; State Key Laboratory of Membrane Biology, Institute of Zoology, Chinese Academy of Sciences, Beijing 100101, China; State Key Laboratory of Membrane Biology, Institute of Zoology, Chinese Academy of Sciences, Beijing 100101, China; University of Chinese Academy of Sciences, Chinese Academy of Sciences, Beijing 100101, China; State Key Laboratory of Membrane Biology, Institute of Zoology, Chinese Academy of Sciences, Beijing 100101, China; University of Chinese Academy of Sciences, Chinese Academy of Sciences, Beijing 100101, China; State Key Laboratory of Membrane Biology, Institute of Zoology, Chinese Academy of Sciences, Beijing 100101, China; Beijing Institute of Genomics, Chinese Academy of Sciences/China National Center for Bioinformation, Beijing 100101, China; University of Chinese Academy of Sciences, Chinese Academy of Sciences, Beijing 100101, China; Beijing Institute of Genomics, Chinese Academy of Sciences/China National Center for Bioinformation, Beijing 100101, China; University of Chinese Academy of Sciences, Chinese Academy of Sciences, Beijing 100101, China; Beijing Institute of Genomics, Chinese Academy of Sciences/China National Center for Bioinformation, Beijing 100101, China; University of Chinese Academy of Sciences, Chinese Academy of Sciences, Beijing 100101, China; State Key Laboratory of Membrane Biology, Institute of Zoology, Chinese Academy of Sciences, Beijing 100101, China; Institute for Stem cell and Regeneration, Chinese Academy of Sciences, Beijing 100101, China; Beijing Institute for Stem Cell and Regenerative Medicine, Beijing 100101, China; University of Chinese Academy of Sciences, Chinese Academy of Sciences, Beijing 100101, China

**Keywords:** carboxypeptidase E, adult neurogenesis, aging, BDNF

## Abstract

Adult neurogenesis declines with age due to the less functional neural stem cells (NSCs) and niches, but the underlying molecular bases for this impaired condition remain unclear. Here we analyzed >55,000 single-cell transcriptomes from two discrete neurogenic niches across the mouse lifespan, and identified new features and populations in NSCs, new markers, and neurogenic regional-specific alternations during aging. Intercellular communication analysis revealed defects in brain-derived neurotrophic factor (BDNF)-TrkB signaling cascade in old NSCs. Carboxypeptidase E (CPE) was found to be highly enriched in NSCs, and played a crucial role in mature/proBDNF balance and adult neurogenesis. Diminishment of CPE with aging resulted in impaired generation of BDNF, thus limiting the neurogenesis in old neurogenic niches. Restoring CPE expression markedly rescued the adult neurogenesis by increasing the production of mature BDNF, offering an attractive therapeutic strategy for the treatment of certain disorders in regions associated with constitutive neurogenesis.

## Introduction

In the adult mammalian brain, neurogenesis mainly occurs in two distinct regions, the subventricular zone (SVZ) in the walls of the lateral ventricles and the subgranular zone (SGZ) in the dentate gyrus (DG) of the hippocampus [1, 2]. The neural stem cells (NSCs) at different stages of commitment reside in a specialized neurogenic niche comprising various types of cells in SVZ or SGZ. The NSCs at different states are regulated by a dynamic cross-talk of both intrinsic determinants and extrinsic niche signals [3], however, the detailed relationship between NSCs and the corresponding niche remains unclear.

Although there is still controversy about the neurogenesis in aging humans [4–8], adult neurogenesis in the SVZ and SGZ of mammals is thought to persist throughout adulthood [2, 7, 9]. Compelling evidence supports that the age-related decline in neurogenesis is attributed to the exhaustion of NSCs and less functional neurogenic niches [10–12]. Although age-dependent changes of the whole neurogenic tissues have been previously studied [13, 14], how change in cell properties and compositions at the single-cell level deteriorates the function of neurogenic niches during aging remains largely unclear.

Recently, single-cell RNA sequencing (scRNA-seq) has been used to study the young [15–24] and old NSC lineages [16, 25, 26], or neurogenic niche [16, 27], which had been focusing on either SVZ [28] or SGZ. Due to the distinct organizations and heterogeneous cellular compositions of these germinal regions, the regulatory network in adult neurogenesis could be complexed by ages, sexes, and brain regions [16, 22, 29]. To fully understand the dynamic and distinct transcriptome changes of each cell type during the declined neurogenesis, a comprehensive and simultaneous single-cell examination of neurogenic niches in both SVZ and SGZ from aging mice is definitely required to fill this gap in knowledge, which, however, is still lacking.

Here, we performed single-cell transcriptome analyses of both SVZ and DG regions in the brains of female and male mice at four different ages. We have identified novel NSC and transient amplifying progenitors (TAP) populations, features of NSC in receptors, markers, and neurogenic regional-specific alternations, and temporal dynamically changed molecules and biological processes with age, providing an aging mechanism for neurogenic regions, and an important resource for the whole neural aging community. Further analyses demonstrated deficiencies of brain-derived neurotrophic factor (BDNF)-TrkB signal cascade in old NSCs, and identified CPE as an effective target for restoring the impaired generation of BDNF and neurogenesis in aged brains. Thus, our findings establish the single cell-based transcriptomic landscapes of neurogenic niches along aging process, and offer a potentially effective therapy to counteract age-related neurological decline in human brains.

## Results

### Distinct single-cell transcriptome profiling of the SVZ and DG regions in adult brains of different sexes and ages

To uncover the single cell-based alterations related to neurogenesis during aging process, the cells in SVZ and DG from one female and one male mice at different ages were subjected to scRNA-seq using the 10× Genomics Chromium platform separately (Fig. S1A, dataset A). For a statistical consideration, we acquired a second dataset (dataset B) with the same experiment setting. Datasets A and B were analyzed independently because they were obtained using different kit versions. After stringent filtering, we obtained 55,166 high-quality cells (28,543 cells for SVZ and 26,623 cells for DG) in dataset A and 99,788 cells (33,068 cells for SVZ and 66,720 cells for DG) in dataset B that were analyzed further. Unless stated otherwise, the follow-up scRNA-seq analysis was focused on dataset A, with some important findings being confirmed using dataset B. For the dynamic changes of cell ratios of different cell types and the sex-related changes, cells were jointly analyzed from all replicates.

**Figure 1.**
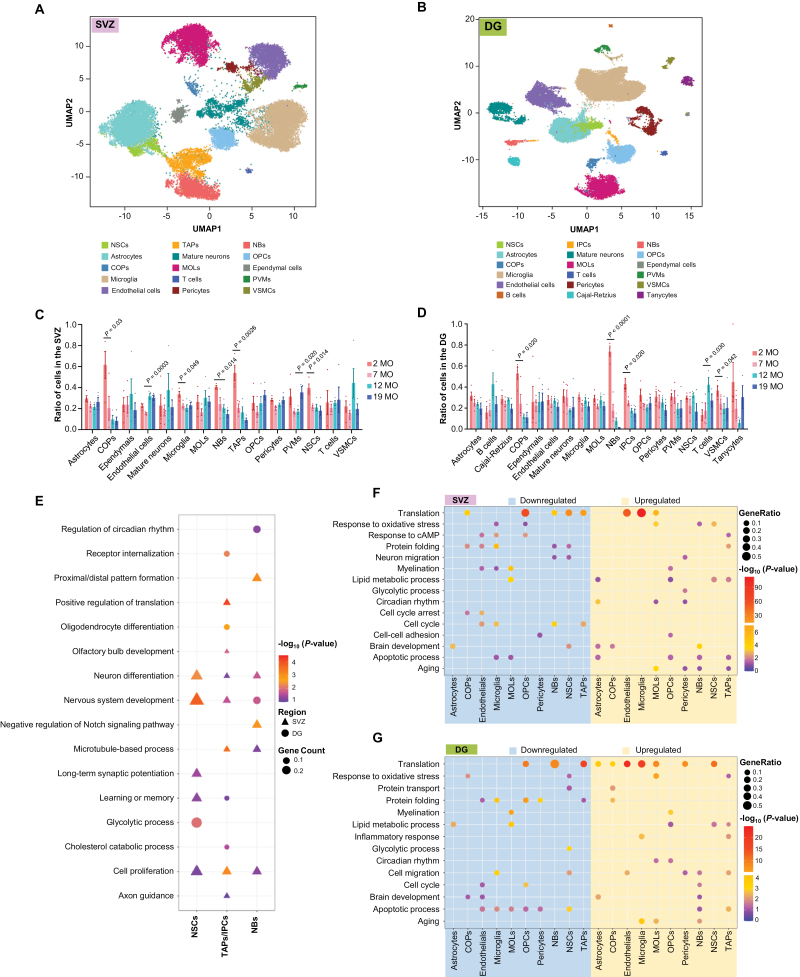
**Profiling and comparison of mouse single-cell atlases of different ages, and brain regions.** (A and B) UMAP plot of the entire SVZ dataset (A, 8 samples, *n* = 28,543 cells, dataset A) and DG dataset (B, 8 samples, *n* = 26,623 cells, dataset A). Cells are colored by different cell types. SVZ, subventricular zone; DG, dentate gyrus; NSCs, quiescent neural stem cells; TAPs, transient amplifying progenitors; NBs, neuroblasts; OPCs, oligodendrocyte progenitor cells; COPs, differentiation-committed oligodendrocyte precursors; MOLs, mature oligodendrocytes; PVMs, perivascular macrophages; VSMCs, vascular smooth muscle cells; IPCs, intermediate progenitor cells. (C and D) For each cell type, bar plot shows the proportion of cells in 16 samples of SVZ (C) and DG (D). Combined analysis of dataset A and B. *n* = four mice each group. *P* values are indicated (one-way ANOVA). (E) Representative GO terms of DEGs of stem cells between SVZ and DG. (F) GO enrichment analysis with DAVID for the DEGs of each cluster in the SVZ between 2 MO and 19 MO. (G) GO Enrichment analysis with DAVID for the DEGs of each cluster in the DG between 2 MO and 19 MO.

The analysis of 55,166 high-quality single-cell transcriptomes from dataset A with UMAP revealed 15 and 18 main cell types in the SVZ and DG, respectively (Fig. 1A and 1B). Cell type assignments for each cluster were generated using multiple known cell-type-specific marker genes and DEGs analysis (Fig. S1B–S1F; Table S2). Specifically, due to the overlapping expression profiles of NSCs and astrocytes (*Aldoc*, *Slc1a3*, *Fabp7*, and *Hes5*), here, NSCs in the SVZ were identified mainly based on some recently reported novel stem cell markers, such as *Nr2e1* [36], *Thbs4* [20], *Igfbp5*, and *Notum* [22]. Moreover, NSCs almost completely lacked expression of many mature astrocyte-specific genes such as *Aqp4*, *Tril*, and *Grin2c* (Fig. S1B). NSCs in the DG did not cluster separately from the astrocytes, and they were identified by the lack of expression of astrocyte marker genes (*Aqp4* and *Grin2c)* and the highly enriched NSC marker genes including *Thrsp*, *Fabp7*, *Hopx*, and *Nr2e1* (Fig. S1D). Replicate profiling of samples showed high concordance (Fig. S1G). Cell counts and other metrics for each cell type from datasets A and B were shown in Fig. S1H. All the clusters from dataset B largely expressed the same enriched markers (Table S2). The identification of NSCs in the SVZ from dataset B was shown as an example (Fig. S1I).

We next analyzed the diversity of cell type distribution during aging and sexes. All cell clusters were shared across four different ages and sexes in each region (Fig. S1J), indicating that the cell number alterations were not big enough to gain or lose a cluster with aging. However, there existed dynamic changes in the cell count in a few cell types. The numbers of TAPs in the SVZ and NBs in the DG showed a drastic and persistent drop from 2 to 19 MO, suggesting that the major decline of neurogenesis in these two regions occurs at different neurogenic stages during aging (Fig. 1C and 1D, joint analysis of datasets A and B).

By analyzing the DEGs of each corresponding cell type between SVZ and DG, we detected significant differences in different stages of stem cells but not in the niche cells (Table S3), suggesting that NSCs, instead of their niches, contribute to the major commitment differences between SVZ and DG.

GO analysis of DEGs revealed the distinct regional features of three types of NSCs (Fig. 1E and Table S4). Relative to DG NSCs, SVZ NSCs highly expressed neuronal differentiation-related genes while lowly expressed glycolytic process-related genes, implying that NSCs from SVZ and DG possess distinct properties in cell differentiation and metabolism (Fig. S2A). Interestingly, we identified much more DEGs between intermediate progenitors in the SVZ and DG (Fig. S2B), supporting a difference in cell division and differentiation direction of proliferating stem cells. Comparison of DEGs of NBs suggests the importance of Notch signaling activity and circadian rhythm regulation in these two regions (Fig. S2C).

To explore the age-related molecular changes, detailed DEGs of each cell type between 2 and 19 MO were identified (Table S5). GO analysis of DEGs revealed the major age-related alterations of biological processes in each cell type (Fig. 1F and 1G). Intriguingly, the “lipid metabolism” pathway was found to be enriched in upregulated genes in aging NSCs of both neurogenic regions, which was in agreement with previous findings that lipid metabolism is involved in NSC aging [37]. As one specific example, we investigated how Apolipoprotein (apo) E, a multifunctional protein with central roles in lipid metabolism, changes with age in the SVZ and SGZ. We found that the expression of APOE increased with age in both neurogenic regions (Fig. S2D).

Interestingly, sex-biased differences in aged NSCs were observed. While some DEGs in aged NSCs were shared in both sexes, 14 genes manifest an opposite alteration pattern between female and male (Fig. S2E and S2F). We further validated the expression of *Prdx1* in NSCs of 2 and 18 MO mice by immunostaining, which showed an age-dependent upregulation in female but not male mice (Fig. S2G and S2H), supporting the sex difference in NSC transcriptome change during aging.

### Two sub-clusters of TAPs showed differences in cell proliferation and DNA damage

**Figure 2.**
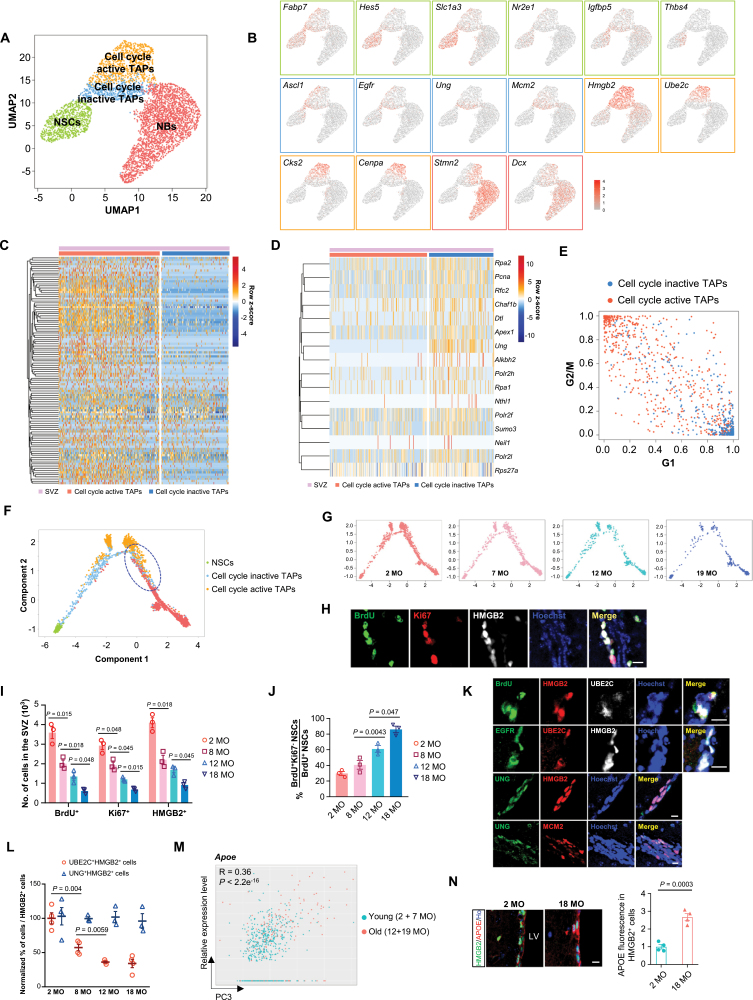
**Classification of cell cycle active and inactive TAPs.** (A) UMAP plot of the NSCs, TAPs, and NBs in the SVZ from dataset A. (B) UMAP visualization of (A) colored by the expression of representative stem cell marker genes. Different color indicates different markers used to identify each cell type. Green: NSCs; blue: cell cycle inactive TAPs; orange: cell cycle active TAPs; red: NBs. (C and D) Heatmap plot for expression of cell cycle-related genes (C) and DNA damage repair-related genes (D) in cell cycle active and inactive TAPs. (E) Cell cycle status (G1 phase and G2/M phase) inference of the cell cycle active and inactive TAPs. (F) Pseudotime trajectory of stem cells in the SVZ colored by different cell types. TAPs at the stage of transition to NBs are circled with blue dashed line. (G) Pseudotime trajectory of stem cells in the SVZ colored by different ages. (H) Immunostaining for BrdU, Ki67, and HMGB2 in the SVZ of 2 MO WT mice. Scale bar, 20 µm. (I) Reduction of TAPs during aging. Quantification of total number of BrdU^+^, Ki67^+^, and HMGB2^+^ cells in the SVZ of 2, 8, 12, and 18 MO mice. Data are represented as mean ± S.E.M. *n* = three mice per age. *P* values are indicated (one-way ANOVA). (J) Quantification of the fraction of proliferating NSCs that exit the cell cycles (BrdU^+^Ki67^-^/BrdU^+^) in the SVZ of 2, 8, 12, and 18 MO mice. Data are represented as mean ± S.E.M. *n* = three mice per age. *P* values are indicated (one-way ANOVA). (K) Representative images of co-staining for UBE2C with BrdU, HMGB2, and EGFR (top two panels) and for UNG with HMGB2 and MCM2 (lower two panels) in the adult SVZ. Scale bars, 10 µm. (L) Quantification of the percentages of UBE2C^+^HMGB2^+^ and UNG^+^HMGB2^+^ cells out of all HMGB2^+^ cells in the SVZ at 2, 8, 12, and 18 MO mice. Data are represented as mean ± S.E.M. *n* = three to four mice per group. *P* values are indicated (one-way ANOVA). (M) Scatter plots showing the relative expression level of one of the top upregulated aging-associated DEG, APOE, in NSCs along PC3 dimension. Each point indicates a single cell. Pearson’s correlation coefficient (*R*) and statistical significance (*P*) are indicated. (N) Representative images of co-staining for APOE with HMGB2 in the TAPs of adult SVZ from 2 MO and 18 MO mice. Ho, Hoechst. Scale bar, 10 µm. Quantification of APOE fluorescence intensity in HMGB2^+^ cells normalized to the SVZ area is shown on the right panel. Data are represented as mean ± S.E.M. *n* = four mice each group. *P* value is indicated (two-tailed *t*-test).

NSCs, TAPs, and NBs were then extracted for further analysis (Fig. 2A and 2B). Different stages of stem cells exhibited similar distribution (Fig. S3A). Further sub-clustering of stem cells led to an identification of two novel subtypes in TAPs (Fig. 2A, dataset A; confirmed by Fig. S3B using dataset B). One subtype is composed of about 60% TAPs with a high expression of cell cycle-related genes, representing a feature of rapid proliferation. The other subtype occupying nearly 40% of TAPs showed a downregulation of cell cycle-related processes and associated genes (Figs. 2B and 2C, S3C–S3E, dataset A; confirmed by Fig. S3F using dataset B). These two subtypes were defined as cell cycle active and inactive TAPs, respectively.

Adjacent to the qNSCs cluster, the population of cell cycle inactive TAPs was found to be enriched for DNA repair-related genes (Fig. 2D), and mostly arrested in G1 phase (Fig. 2E), which are two features of aNSCs reported by previous scRNA-seq [18, 26], suggesting that they were mostly consisted of activated NSCs. However, some of these cell cycle inactive cells might also include TAPs that are at the stage of transition to NBs or proliferating NSCs that return to quiescence. By contrast, the cell cycle active TAPs, which are close to the tip of the NB cluster, showed a high expression of G2/M genes (Fig. 2E) and increased expression of neuronal marker genes, representing a high potency of differentiation (Fig. 2B). Using pseudotime analysis, we found that qNSCs enter cell cycle to become aNSCs and give rise to actively proliferating TAPs, followed by differentiation into NBs (Fig. 2F), which confirms the linear neurogenic lineage progression of NSCs. Notably, the NSCs and their progeny at four different ages shared the same lineage progression (Fig. 2G). The sequential gene expressions, including *Egfr*, *Ung*, *Ube2c*, *Stmn2*, and *Dcx*, were then evaluated using pseudotime analysis, and the findings further confirmed the lineage specification of NSCs (Fig. S3G).

Subclustering of IPCs in the DG also identified two subtypes (Fig. S3H, combined analysis of datasets A and B). Consistent with the SVZ data, about 60% of IPCs show a high expression of cell cycle-related genes. Importantly, Ube2c could also be used as a marker to distinguish these two subtypes of IPCs (Fig. S3I). These results suggest that there exist some common molecular features of proliferating NSCs in both neurogenic niches.

We then investigated the dynamic changes of the two subtypes of TAPs during aging. The cell count of both subtypes exhibited a sharp decline from 2 to 19 MO (Fig. S3J). Proliferating NSCs were monitored by BrdU together with Ki67 and HMGB2. Almost all BrdU^+^ and Ki67^+^ cells are HMGB2^+^ in both SVZ (Fig. 2H) and DG (Fig. S3K), indicating that HMGB2 can be used as a novel marker for proliferating NSCs. The number of BrdU^+^, Ki67^+^, and HMGB2^+^ cells were progressively decreased along aging (Fig. 2I), while the fraction of proliferating NSCs that exited the cell cycle (BrdU^+^Ki67^-^) increased gradually and substantially with age (Fig. 2J). Interestingly, HMGB2^+^ cells can be further subdivided into two distinct populations, UBE2C^+^HMGB2^+^ and UNG^+^HMGB2^+^. The fast-proliferating UBE2C^+^HMGB2^+^ cells were BrdU^+^ and EGFR^+^, while the UNG^+^HMGB2^+^ cells were MCM2^+^ (a G1 marker which can be detected after a cell has excited the cell cycle) (Fig. 2K). The proportion of UNG^+^HMGB2^+^ cells among all HMGB2^+^ cells remained constant with age, while the UBE2C^+^HMGB2^+^ cells was significantly decreased in aged mice (Fig. 2L), indicating that it is the reduction of proliferative activity of TAPs that primarily contributes to decreased neurogenesis in aged mice.

Additionally, DEG analysis between young and old TAPs in the SVZ showed that *Apoe* was one of the top upregulated aging-associated DEGs (Fig. 2M). There was a significantly higher expression of APOE in HMGB2^+^ TAPs of 18 MO mice compared to that of 2 MO mice (Fig. 2N). Therefore, *Apoe* could be involved in the regulation of TAP aging.

### Ligand-receptor interaction analysis reveals the age-dependent changes in intercellular communication of NSCs and their niches

Except for the age-dependent changes of gene expression in NSCs, the less functional niches are also responsible for the age-related neurogenesis waning. We then investigated the cell-cell interaction network among the cell types identified in the SVZ or DG. Compared with other cell types in the SVZ, astrocytes, and oligodendrocytes showed more interactions with NSCs and TAPs (Fig. S4A).

We then inferred global intercellular communications between NSCs and total cells (see Supplementary Methods). Our results showed that ligands derived from OPCs, NSCs, astrocytes, TAPs, MOLs, endothelial cells, and pericytes had a greater communication potential than other cell types to interact with receptors expressed by NSCs, while ligands derived from NSCs appeared to communicate more with receptors from OPCs, astrocytes, and TAPs compared to other cell types in the SVZ (Fig. S4B).

**Figure 3.**
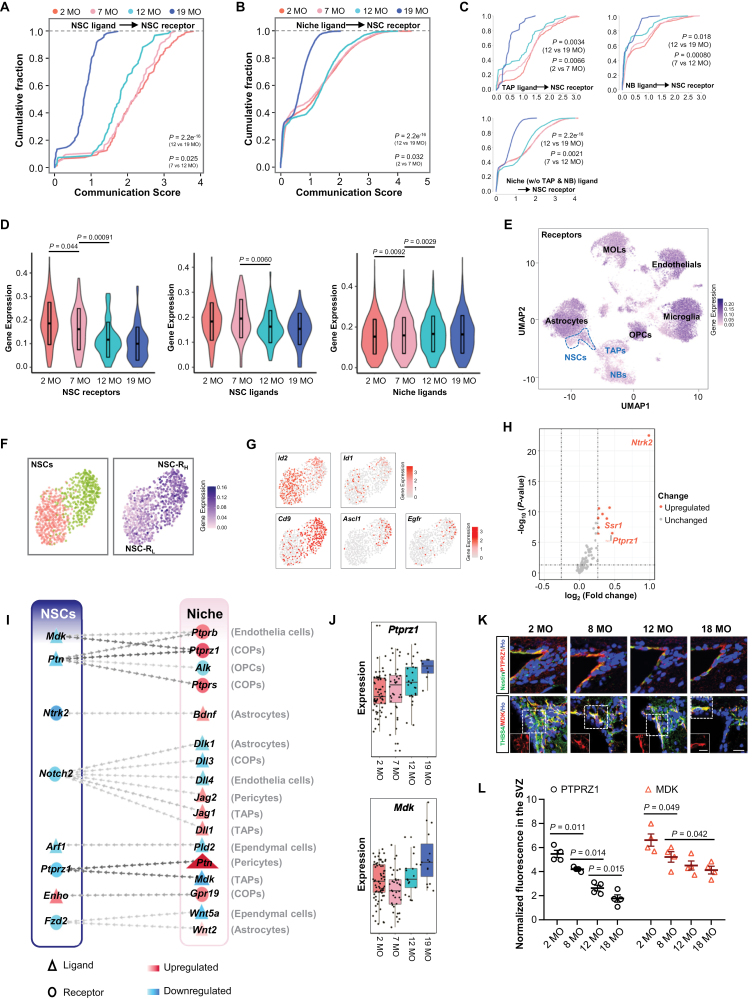
**Cell-to-cell communication analysis and ligand/receptor influences calculation in the SVZ.** (A) Cumulative fraction of each NSC in the SVZ according to global communication scores between NSC ligands and NSC receptors. (B) Cumulative fraction of each niche cell (including TAP, NB, and other cell types in the SVZ) in the SVZ according to global communication scores between niche ligands and NSC receptors. (C) Cumulative fraction of each TAP/NB/niche cell without TAP and NB in the SVZ according to global communication scores between niche ligands and NSC receptors. (D) Violin plots with data in TPM showing ligands/receptors expression levels for each age. The expression of receptors/ligands in each cell at each age (two mice at each age) was used. Ligands are those correspond to receptors expressed by NSCs. Data are represented as mean ± S.D. *P* values are indicated (one-way ANOVA). (E) Expression plot of receptors involved in ligand-receptor interactions over the UMAP map to assess ligand-receptor interaction prevalence in the SVZ. Outlined region indicates the NSC cluster. (F) Analysis about heterogeneity of NSCs in the SVZ based on the receptor expressions. Subclustering analysis using unsupervised hierarchical clustering of NSCs revealed two subclusters (left). UMAP plot of NSCs showing the expression of receptors involved in ligand-receptor interactions (right). (G) UMAP visualization of (F) colored by the expression of NSC quiescence-related marker genes (*Id2* and *Id1*) and genes involved in activation of qNSCs (*Cd9*, *Ascl1*, and *Egfr*). NSC-R_H_ show higher expression of activation markers. (H) Volcano plot of differentially expressed receptor genes between NSC-R_H_ and NSC-R_L_ in the SVZ. Top leading significant receptors are labeled by gene names. (I) Aging-related changes in intercellular communication in the SVZ. Triangles and ellipses indicate ligands and receptors, respectively. The differentially expressed ligands and receptors in NSCs (left column) and the corresponding receptors or ligands in the niche (right column) are listed on columns. Dot colors means the fold change of each gene, while red for up-regulated genes in 19 MO and blue for down-regulated in 19 MO. Ligands and receptors that interact are lined by a chain of arrows, the color of which means the nMI of the given ligand and receptor. The exact niche cell types that interact with NSCs for each ligand or receptor were shown on the right. (J) Boxplot for the expression of *Ptprz1* and *Mdk* in the NSCs of the SVZ that do express the gene at each age group. (K) Representative images of co-staining for PTPRZ1/MDK with NSC markers (THBS4 and Nestin) in the NSCs of adult SVZ from mice at 2, 8, 12, and 18 MO. Ho, Hoechst. Scale bars, 20 µm. (L) Normalized PTPRZ1 and MDK fluorescence intensity in the SVZ of mice at 2, 8, 12, and 18 MO, respectively. Data are represented as mean ± S.E.M. Each dot represents mean normalized protein fluorescence in three sections from one mouse. *n* = four mice each age. *P* values are indicated (one-way ANOVA).

Compared to niche cells, ligands secreted by NSCs showed much stronger communication influence on NSC receptors. Moreover, more than 30% of niche cells showed no communication to NSCs. The communication between NSC-secreted ligands and NSC receptors decreased significantly with age (Fig. 3A), whereas the communication between niche (including TAPs, NBs, and other cell types in the SVZ) ligands and NSC receptors did not show a significant decrease until 19 MO (Fig. 3B). Of note, among all the niche cells, ligands secreted by TAPs showed much stronger communication influence on NSC receptors compared to NBs and other cell types in the SVZ (Fig. 3C). These data reveal the strong communication between NSC-secreted ligands and NSC receptors, which is also mostly affected by aging.

The expression of NSC receptors and their corresponding NSC ligands showed an overall decreased trend with age. By contrast, the niche ligands interacting to NSC receptors showed an increased trend with age (Fig. 3D). These results indicate that the decreased expression of NSC receptors and/or NSC ligands could be the main cause of the attenuated ligand-receptor communication during aging, which may highly associate with the age-related decline of neurogenesis in SVZ.

To assess ligand-receptor interaction prevalence, we also plotted the expression of receptors (Fig. 3E) and ligands (Fig. S4C). Notably, in the NSCs cluster, we observed that receptor genes were highly expressed at the right part of the cluster, but lowly expressed at the left part (Fig. 3E). Further subclustering of NSCs by unsupervised clustering indeed obtained two subclusters (Fig. 3F). We therefore named these two populations as NSC-R_H_ and NSC-R_L_, respectively, based on the relative expression of receptor genes.

DEG analysis between NSC-R_H_ and NSC-R_L_ showed that many protein-synthesis-associated genes as well as cell cycle-related genes were upregulated, while quiescence-associated genes were downregulated in NSC-R_H_ (Fig. S4D), consistent with molecular features of primed qNSCs reported previously [20, 23]. Therefore, we propose that these NSC-R_L_ and NSC-R_H_ were dormant-NSCs and primed-qNSCs, respectively. In support of that, using recently reported markers for dormant and primed qNSCs [38], we found that *Id2* and *Id1*, hallmark genes of quiescence, are highly expressed in the NSC-R_L_ whereas *Cd9, Ascl1*, and *Egfr*, genes involved in activation of qNSCs, are highly expressed in NSC-R_H_ (Fig. 3G). Similar subclustering of NSCs were obtained in the DG (Fig. S4E). Most of findings presented above were confirmed using the separate dataset B (Fig. S4F–H). Of note, the proportion of NSC-R_H_ was found to be decreased with age, whereas NSC-R_L_ showed an increase in ratio during aging (Fig. S4I), indicating the exhaustion of primed-qNSCs (NSC-R_H_) with age.

We found that around 80% of SOX2^+^GFAP^+^ cells showed THBS4 expression, consisting with the observation that SVZ NSCs are mostly quiescent. Notably, a subset of THBS4-expressing qNSCs, in which nestin was negative or lowly expressed, expressed EGFR, a widely used marker for aNSC (Fig. S4J), suggesting the existence of a small population of qNSCs that are pre-activated before they are activated to generate proliferative TAPs. We propose that THBS4^+^EGFR^+^ could be used to distinguish the primed qNSCs from dormant qNSCs (THBS4^+^SOX2^+^GFAP^+^) and aNSCs (Thbs4^-^EGFR^high^Nestin^high^).

Among the upregulated receptor genes, *Ntrk2* (encoding TrkB) was one of the top leading genes in the NSC-R_H_ compared to NSC-R_L_ from both neurogenic regions (Figs. 3H and S4K). Among the THBS4^+^ cells, TrkB were mostly detected in THBS4^+^EGFR^+^ cells (Fig. S4L). These results strongly support an idea that expressions of certain receptor genes are important for the mobilization of qNSCs.

We then constructed a ligand-receptor expression network between NSCs and their niches in the SVZ (Fig. 3I) and DG (Fig. S4M). Remarkably, *Ntrk2* and *Ptprz1* were the most significantly downregulated receptors during aging. Consistent with *Ptprz1* and *Mdk* expression pattern (Fig. 3J), the overall PTPRZ1 and MDK protein levels was reduced during aging, which was mainly due to the significantly less abundant of qNSCs in old mice, although the remaining MDK/PTPRZ1-positive cells were more intensely labeled compared to the young brain (Fig. 3K and 3L). These observations indicate that changes of these ligand-receptor interactions may play crucial roles in aging-related decline of neurogenesis.

### Aging causes deficiency of TrkB pathway and defect in BDNF generation in the SVZ

**Figure 4.**
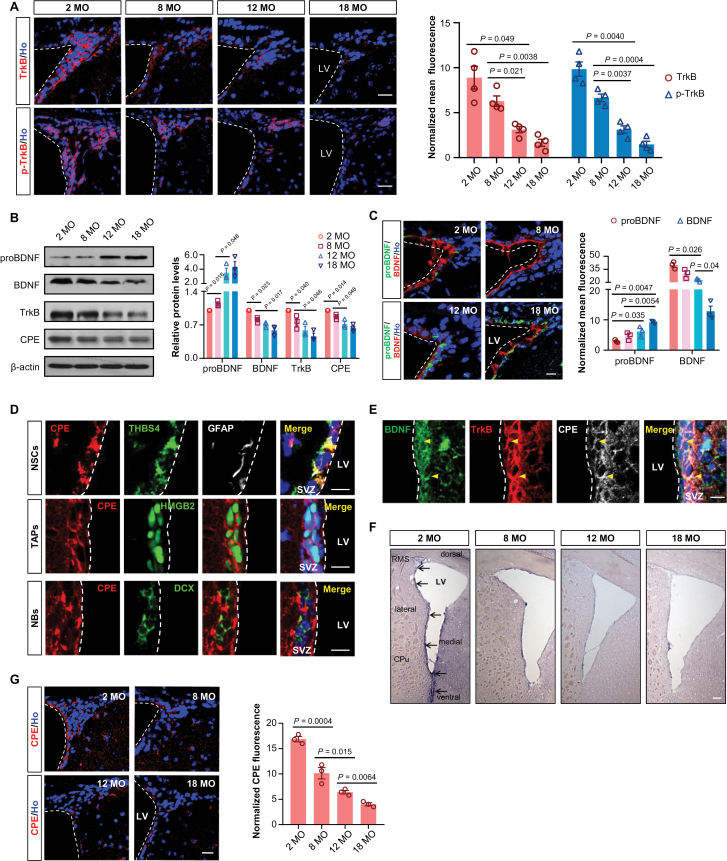
**Aging causes deficiency of BDNF-TrkB pathway and defect in CPE in the SVZ.** (A) Representative images (left) and normalized fluorescence intensity (right) of TrkB and p-TrkB in the SVZ of mice at 2, 8, 12, and 18 MO. Ho, Hoechst. Scale bars, 20 µm. Data are represented as mean ± S.E.M. *n* = four mice each age. (B) Western blotting analyses (left) and relative quantification of expression level (right) of proteins extracted from the mixed tissues of SVZ from three mice at each age. β-actin is used as a loading control. Data are represented as the mean protein intensity normalized to β-actin ± S.E.M. *n* = three mice each age. (C) Representative images (left) and normalized fluorescence intensity (right) of proBDNF and BDNF expression in the SVZ of mice at 2, 8, 12, and 18 MO. Scale bars, 20 µm. Data are represented as mean ± S.E.M. *n* = three mice each age. (D) Characterization of CPE-positive NSCs (THBS4^+^GFAP^+^, top panel), TAPs (HMGB2^+^, middle panel), and NBs (DCX^+^, bottom panel) in the SVZ of 2 MO mice. Scale bars, 20 µm. (E) Representative picture of co-staining for BDNF, TrkB, and CPE in the SVZ. Scale bars, 10 μm. Yellow arrowheads indicate TrkB^+^CPE^+^BDNF^+^ cells. (F) *In situ* hybridization analysis of *CPE* mRNA in the SVZ of mice at 2, 8, 12, and 18 MO. Arrows indicate positive signals. Scale bars, 100 µm. RMS, rostral migratory stream; LV, lateral ventricles; Cpu, caudate putamen. (G) Representative images of CPE expression (Left) and its normalized fluorescence intensity (Right) in the SVZ of mice at 2, 8, 12, and 18 MO. Scale bars, 20 µm. Data are represented as mean ± S.E.M. *n* = three mice each age. P values are indicated (one-way ANOVA).

Our ligand-receptor analysis revealed that TrkB (encoded by *Ntrk2*) pathway of NSC was significantly affected during aging. Given that the SVZ is the largest neurogenic region in adult brain, the following study was focused on the SVZ. Expression of TrkB and p-TrkB (the endogenous activated form of TrkB) was detected in all main stages of NSC-lineages (Fig. S5A and S5B). In line with *Ntrk2* pattern of expression (Fig. S5C), TrkB and p-TrkB expression gradually decreased with age (Fig. 4A and 4B), indicating that the decline of TrkB signaling cascade might contribute to the age-induced reduction of adult neurogenesis.

We then analyzed the expression patterns and dynamic changes of two TrkB ligands, BDNF and neurotrophin-4. Both neurotrophic factors showed local enrichment in the SVZ (Fig. S5D). BDNF was specifically expressed in Nestin^+^GFAP^+^ NSCs, HMGB2^+^ TAPs, and PSA-NCAM^+^ NBs (Fig. S5E). Furthermore, we observed a consistent age-related decline in the expression of mature BDNF (mBDNF). Surprisingly, proBDNF, the precursor form of BDNF, exhibited an age-related increase (Fig. 4B and 4C). The imbalance between mBDNF and proBDNF indicates a defect in mBDNF generation in the SVZ of aged brains.

To identify which factor regulates the processing of BDNF during aging, we analyzed the DEGs of NSCs between 2 and 19 MO. Intriguingly, *Cpe* gene encoding a member of metallocarboxypeptidases [39–44], was found to be significantly downregulated in aged mice (Fig. S5F). Importantly, CPE was previously reported to be required for both cellular transport and secretion of BDNF [44, 45].

The effects of CPE depend on the localization of its expression [41, 46–48], and the expression pattern of CPE in the adult neurogenic regions and across the lifespan are not clear yet. Here, CPE was found to be expressed in all stages of NSCs in the SVZ (Fig. 4D), and the co-localization of TrkB, BDNF, and CPE was further confirmed by triple immunostaining (Fig. 4E). In agreement with the linear decline of *Cpe* expression pattern with age (Fig. S5G), we found that the mRNA transcripts (Fig. 4F) and the protein expression levels (Fig. 4B and 4G) of CPE declined progressively with age. Given the important role of CPE in the transport and secretion of BDNF [40, 43, 45], we speculate that insufficient CPE could be the reason for the defective BDNF generation in the SVZ of aged brains.

### CPE promotes the adult neurogenesis in the SVZ via BDNF-TrkB signaling pathway

**Figure 5.**
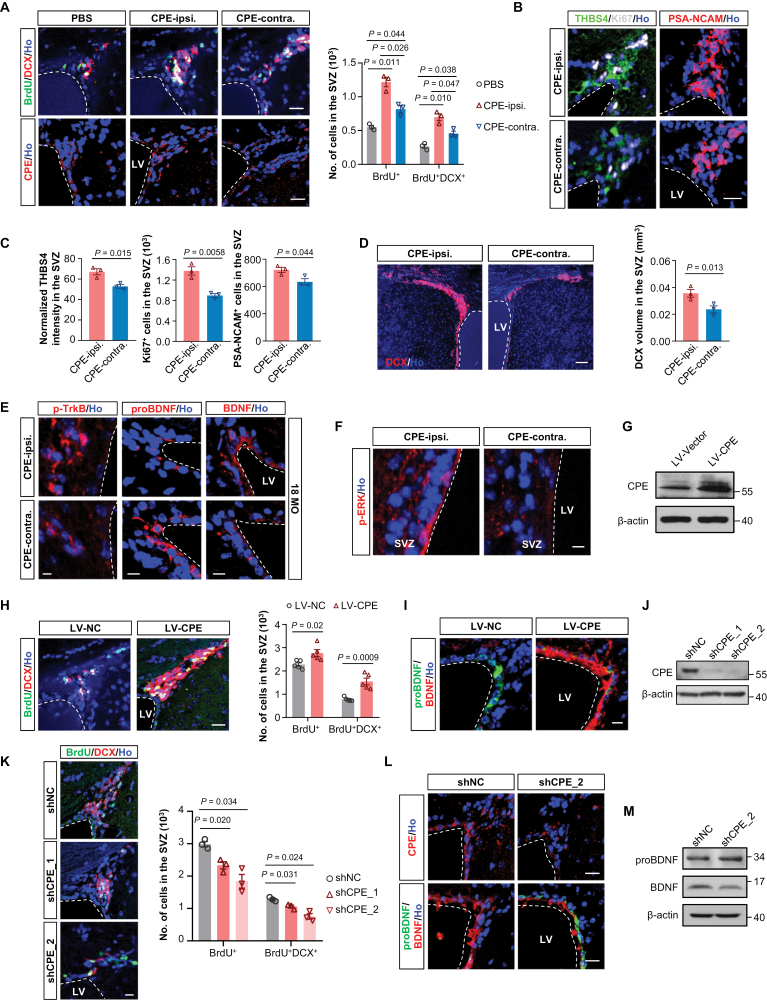
**CPE promotes the adult SVZ neurogenesis through the BDNF-TrkB signaling pathway.** (A) Left, representative images of BrdU and DCX double-labelled newly generated neurons (top panel) or CPE staining (bottom panel) in the SVZ of 18 MO mice one week after PBS or CPE infusion. Ho, Hoechst. Scale bars, 10 µm. ipsi., ipsilateral, the injection side; contra., side contralateral to CPE injection. Quantification of BrdU^+^ and BrdU^+^DCX^+^ cells in the SVZ was shown on the right. Data are represented as mean ± S.E.M. *n* = three mice per group. *P* values are indicated (one-way ANOVA). (B) Images of THBS4 (radial glia-like and projenitor cells marker, left panel), Ki67 (proliferating cells marker, left panel), and PSA-NCAM (immature neuron marker, right panel) expression demonstrate increased proliferation and neuronal differentiation in the ipsilateral SVZ compared with contralateral SVZ from 18 MO mice one week after CPE infusion. Scale bars, 20 µm. (C) Normalized THBS4 fluorescence intensity and quantification of numbers of Ki67^+^ and PSA-NCAM^+^ cells in (B). Data are represented as mean ± S.E.M. *n* = three mice per group. *P* values are indicated (two-tailed *t*-test). (D) DCX volume is significantly increased in the ipsilateral SVZ compared with contralateral SVZ from 18 MO mice one week after CPE infusion. Scale bars, 100 µm. Quantification of the DCX volume was shown on the right panel. Data are represented as mean ± S.E.M. *n* = three mice per group. *P* values are indicated (two-tailed Student's t test). (E and F) Representative images of p-TrkB, proBDNF, and BDNF (E) and p-ERK (F) expression in the ipsilateral SVZ compared with contralateral SVZ from 18 MO mice one week after CPE infusion. Scale bars, 10 µm. (G) Western blotting analysis of CPE expression levels in the SVZ of mice injected with lentiviruses expressing NC (LV-NC) or wild-type CPE (LV-CPE). β-actin is used as a loading control. (H) Left, Representative images of BrdU and DCX double-labelled newly generated neurons in the SVZ of mice with grafted LV-NC or LV-CPE. Quantification of the number of BrdU^+^ and BrdU^+^DCX^+^ cells was shown on the right. Data are represented as mean ± S.E.M. *n* = five mice each group. *P* values are indicated (two-tailed *t*-test). (I) Representative images of proBDNF and BDNF expression in the SVZ with grafted lentivirus expressing LV-NC or LV-CPE. Scale bars, 10 µm. (J) Western blot analysis of the efficacy of the shRNA against mouse CPE in N2a cells. β-actin is used as a loading control. (K) Left, Representative images of BrdU and DCX double-labelled newly generated neurons in the SVZ of middle-aged mice injected with lentiviruses expressing shNC, shCPE_1, or shCPE_2. Quantification of the number of BrdU^+^ and BrdU^+^DCX^+^ cells in the SVZ was shown on the right. Data are represented as mean ± S.E.M. *n* = three mice each group. *P* values are indicated (one-way ANOVA). (L) Representative images of CPE, proBDNF, and BDNF expression in the SVZ of mice injected with lentiviruses expressing shNC or shCPE_2. Scale bars, 10 µm. (M) Western blotting analyses of proteins extracted from the mixed tissues of SVZ from three mice injected with lentiviruses expressing shNC or shCPE_2. β-actin is used as a loading control.

Despite the neurotrophic roles of CPE in NSC differentiation and neuroprotection have been reported [41, 48, 49], the role of CPE in adult neurogenesis has not been fully explored. CPE is a secreted protein, which can be efficiently secreted and internalized to lysosomes of neighboring cells [50]. To explore the role of CPE in regulating adult neurogenesis *in vivo*, we performed an infusion of CPE into the left lateral ventricle of aged (18 MO) mice. Neurogenesis and CPE levels in the ipsilateral SVZ of CPE-treated mice increased significantly compared with the contralateral SVZ or with vehicle-treated mice (Figs. 5A and S6A), supporting the internalization of infused CPE to NSCs, which further promotes neurogenesis. Exogenous infusion of CPE led to a robust increase in THBS4^+^ NSCs and progenitor cells, Ki67^+^ proliferating cells as well as PSA-NCAM^+^ immature neurons (Fig. 5B and 5C), indicating that replenishment of CPE expands adult NSC and progenitor pool, which was accompanied by a significantly enhanced proliferation of NSCs and the subsequent neuronal differentiation.

Consistent with the NSC expansion, the DCX volume in the ipsilateral SVZ increased significantly compared with the contralateral side (Fig. 5D). In addition, we found that CPE replenishment resulted in an increase in glia produced in both the NSCs and the niche in the ipsilateral SVZ (Fig. S6B), suggesting that CPE in some way promotes a neuronal cell fate to some extent but this is not necessarily at the expense of glial differentiation.

Exposure to exogenous CPE in aged mice led to a significantly higher levels of p-TrkB and mBDNF but a lower level in proBDNF in NSCs of ipsilateral SVZ compared with the contralateral SVZ (Figs. 5E and S6C), indicating that CPE infusion enhances mBDNF levels and activates TrkB. Further, because phosphorylation of the TrkB^Y515^ residue leads to the activation of the MAPK/ERK cascade, we also investigated whether this pathway is involved. A significant increase in the expression level of phosphorylated ERK (p-ERK) in the ipsilateral SVZ was observed (Figs. 5F and S6D), indicating active BDNF-TrkB-ERK signalings in NSCs and progenitor cells.

We also evaluated the effect of exogenous infusion of CPE on the neurogenesis in middle-aged mice. Using 9 MO mice as an example, a similar tendency for enhanced neurogenesis, NSC proliferation, and neuronal differentiation as well as an imbalance of mBDNF and proBDNF was observed (Fig. S6E–H), suggesting that CPE infusion can boost neurogenesis regardless of mice ages.

To further support these findings, we stereotaxically injected lentiviruses expressing negative control (NC) or wild-type CPE (Fig. 5G) into the lateral ventricle of mice. One week after injection, LV-CPE-expressed mice displayed significantly enhanced neurogenesis and levels of mBDNF in the SVZ relative to LV-NC-expressed mice (Figs. 5H, 5I and S6I), indicating that replenishment of CPE, either by exogenous infusion or by overexpression, promotes neurogenesis, which might be due to the elevated mBDNF levels.

Middle-aged mice were used to evaluate whether knockdown of CPE affected neurogenesis *in vivo*. Two different shRNAs against the mouse CPE gene were designed and their silencing effect was demonstrated in N2a cells (Fig. 5J). We found that knockdown of CPE impaired SVZ neurogenesis and decreased mBDNF levels (Figs. 5K–M, S6J, and S6K), indicating that CPE is a potent positive regulator in BDNF generation and SVZ neurogenesis.

### CPE upregulates mBDNF levels through promoting the cleavage efficiency of convertases in NSCs

**Figure 6.**
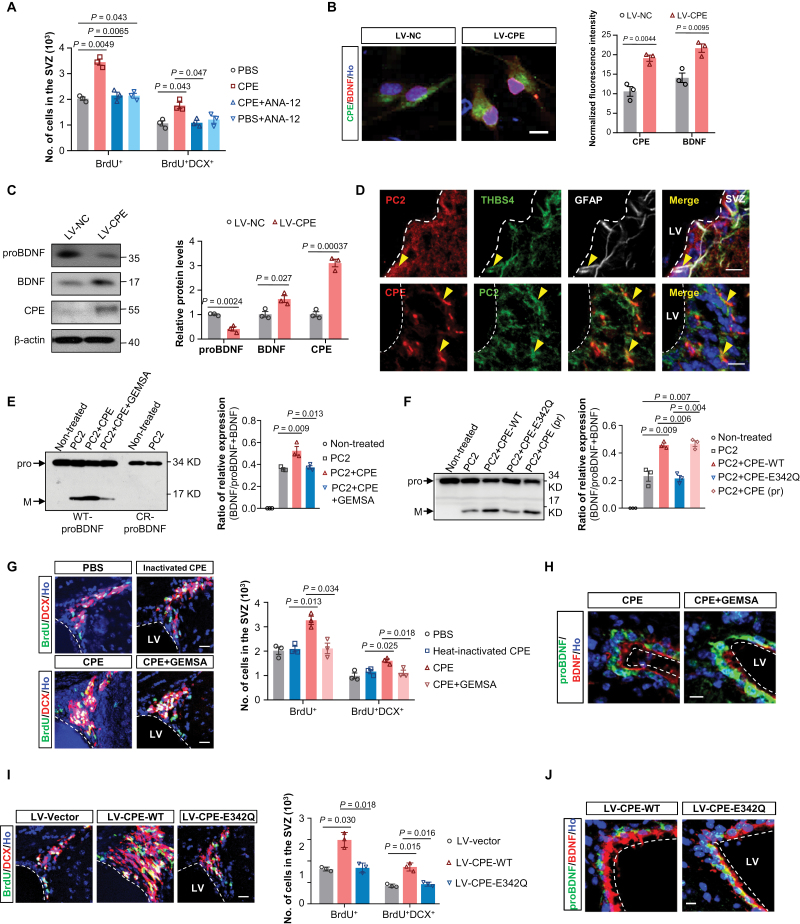
**CPE upregulates BDNF levels through promoting the cleavage efficiency of convertases in NSCs.** (A) Quantification of BrdU^+^ and BrdU^+^DCX^+^ cells in the SVZ of PBS- or CPE-infused mice with or without the TrkB antagonist ANA-12 treatment. Data are represented as mean ± S.E.M. *n* = three mice each group. *P* values are indicated (one-way ANOVA). (B) Representative immunofluorescence images of primary NSCs stained with CPE and BDNF and Hoechst after LV-NC/CPE infection at adherent conditions. Ho, Hoechst. Scale bar, 10 μm. Normalized CPE and BDNF fluorescence intensity was shown on the right. Data are represented as mean ± S.E.M. *n* = three independent experiments. *P* values are indicated (Student’s *t* test). (C) Western blotting analyses of proteins extracted from the primary NSCs 48 h after LV-NC or LV-CPE infection. β-actin is used as a loading control. Relative quantification of Western blotting analysis of protein levels was shown on the right. Data is represented as the mean protein intensity normalized to actin ± S.E.M. from three independent experiments. *P* values are indicated (two-tailed *t-*test). (D) Representative image of co-staining for PC2, THBS4, and GFAP (top panel) in the SVZ and for colocalization of CPE and PC2 (bottom panel) in NSCs in the SVZ. Scale bars, 10 μm. Yellow arrowheads indicate PC2^+^THBS4^+^GFAP^+^ or CPE^+^PC2^+^ cells. (E) Western blotting analyses (left) and relative quantification of the fraction of BDNF/(proBDNF + BDNF) (right) by incubating commercial recombinant wild-type proBDNF (WT-proBDNF) or cleavage-resistant proBDNF (CR-proBDNF) with PC2, CPE, and GEMSA *in vitro*. Bar graphs represent the mean ± SEM from three independent assays. *P* values are indicated (one-way ANOVA). (F) Western blotting analyses (left) and relative quantification of the fraction of BDNF/(proBDNF + BDNF) (right) by incubating commercial recombinant proBDNF with PC2, recombinant CPE-WT, recombinant CPE-E342Q or commercial CPE protein (CPE pr) *in vitro*. Bar graphs represent the mean ± SEM from three independent assays. *P* values are indicated (one-way ANOVA). (G) Left, Representative images of BrdU and DCX double-labelled newly generated neurons in the SVZ of PBS-, heat-inactivated CPE-(top panel), or native CPE-infused mice with or without GEMSA treatment (bottom panel). Quantification of BrdU^+^ and BrdU^+^DCX^+^ cells in the SVZ was shown on the right. Data are represented as mean ± S.E.M. *n* = three mice each group. *P* values are indicated (one-way ANOVA). (H) Representative images of proBDNF and BDNF expression in the SVZ of CPE-infused mice with or without GEMSA. Scale bar, 10 μm. (I) Left, representative images of BrdU and DCX double-labelled newly generated neurons in the SVZ of mice with grafted LV-vector, LV-CPE-WT, or LV-CPE-E342Q. Quantification of BrdU^+^ and BrdU^+^DCX^+^ cells in the SVZ was shown on the right. Data are represented as mean ± S.E.M. *n* = three mice each group. *P* values are indicated (one-way ANOVA). (J) Representative images of proBDNF and BDNF expression in the SVZ of mice with grafted LV-CPE-WT and LV-CPE-E342Q. Scale bar, 10 μm.

Next, we want to understand how CPE leads to increased neurogenesis. To investigate the functional roles of TrkB in this process, we used ANA-12, a selective TrkB antagonist, to inhibit the activation of TrkB. Injection of ANA-12 abolished the increased neurogenesis induced by exogenous infusion of CPE (Fig. 6A), suggesting that the activation of TrkB is required for the boosted CPE-mediated neurogenesis.

To dissect the direct influence of CPE on the regulation of BDNF-TrkB signaling within the NSC lineages, we performed an *in vitro* analysis in cultured NSCs isolated from the SVZ (Fig. S7A). Ectopic expression of CPE significantly increased the BDNF levels in cultured NSCs (Fig. 6B and 6C), consistent with that observed *in vivo*, supporting that CPE can regulate the BDNF-TrkB signaling in NSCs.

Next, we investigated the cell biological mechanism by which CPE might upregulate mBDNF levels. In the mammalian brain, BDNF is initially synthesized as a precursor (proBDNF) [51, 52]. Intracellular cleavage of proBDNF occurs either in the trans-Golgi by endopeptidases, or in secretory granules by pro-protein convertase1/3 [53]. ProBDNF can also be cleaved extracellularly by extracellular proteases such as plasmin and matrix metalloproteases [34, 54, 55]. CPE functions after endopeptidases which removes basic residues from the COOH termini of peptide intermediates. So far, whether PC2, one main endopeptidase that works best at acidic pH like CPE, could cleave proBDNF is unclear.

We examined the expression of several peptide precursor processing enzymes in the SVZ, including furin, PC2, MMP-9, and tPA and its direct target, plasminogen, which can be proteolytically cleaved by tPA to form plasmin. All these enzymes were expressed in the NSCs and their progeny (Fig. S7B). Of note, PC2 was localized in THBS4 and GFAP double-positive radial glia-like NPCs and showed a strong colocalization with CPE in the SVZ (Fig. 6D).

We next performed biochemical analysis *in vitro* to assess the influence of CPE on their capability to cleave proBDNF. Consistent with previous reports, furin, PC1/3, PC2, MMP-9, or plasmin alone could cleave proBDNF. Interestingly, although CPE itself was unable to cleave proBDNF, the incubation of proBDNF with CPE together with PC2, or MMP-9, or plasmin, but not furin or PC1/3, produced a uniform increase of the ratio of mBDNF to proBDNF, despite the observed variability in the promoting extent (Fig. S7C). These data suggest that CPE affects proBDNF cleavage indirectly.

We then selected PC2 for further analyses due to its strong colocalization with CPE and their similar optimum pH. GEMSA, a specific inhibitor of CPE enzymatic activity, was used. We found that GEMSA significantly inhibited the promoting effect of CPE on PC2 cleavage of proBDNF (Fig. 6E), suggesting that CPE promotes the PC2 cleavage efficiency in an enzymatic-dependent manner. When using a proteolytic cleavage-resistant proBDNF mutant (CR-proBDNF, with mutations at R129A and R130G) as a substrate, no mBDNF signal was observed after PC2 incubation, suggesting that mutations of R129 and R130 will perturb the cleavage of proBDNF by PC2 *in vitro*.

To further validate these results, we constructed an enzymatically inactive mutant form of CPE, CPE-E342Q. CPE-WT and CPE-E342Q were expressed at comparable levels (Fig. S7D), with the recombinant mutant showing virtually no detectable enzyme activity (Fig. S7E). PC2 plus CPE treatment, either recombinant or commercial protein, significantly elevated the ratio of mBDNF to proBDNF, with no differences between these two groups, while the recombinant CPE-E342Q had no effect on the PC2-induced cleavage of proBDNF (Fig. 6F), suggesting that CPE contributes positively to the PC2-induced cleavage, which are associated with its enzymatic activity.

We next assessed whether CPE regulates neurogenesis through the activation state of CPE. We denatured CPE from commercial sources *in vitro* by heating at 100°C for 5 min and then grafted this heat-inactivated CPE into the lateral ventricle of mice. Interestingly, we found that infusion of heat-inactivated CPE or co-infusion of CPE with GEMSA abrogated the promoting effects of exogenous native CPE on the neurogenesis, which was accompanied by decreased mBDNF compared to the CPE alone infusion (Figs. 6G, 6H and S7F). We therefore hypothesized that this action of CPE might be associated with its enzymatic activity.

To assess this possibility, we assessed the influence of the enzymatic inactive mutant CPE-E342Q on adult neurogenesis *in vivo*. Relative to CPE-WT-expressed mice, CPE-E342Q-expressed mice displayed decreased neurogenesis and a significantly lower level of mBDNF in the SVZ (Figs. 6I, 6J and S7G), demonstrating that CPE upregulates BDNF levels and promotes neurogenesis in the SVZ in an enzymatic activity-dependent manner.

## Materials and methods

### Ethics statement

All experimental procedures were in full compliance with the Institute of Zoology’s Guidelines for the Care and Use of Laboratory Animals. The experimental protocols were approved by the Animal Care and Use Committee at the Institute of Zoology, Chinese Academy of Sciences (Permission Number: IOZ18009, 8 March 2018).

### Animals

For single-cell analysis, 16 wild-type female and male C57BL/6J adult mice at 2, 7, 12, and 19 months old (MO) were used. All animals were housed in temperature- and humidity-controlled rooms with *ad libitum* access to food and water.

### Cell lines

293T and N2a cells were cultured in DMEM medium supplemented with 10% FBS at 37°C in 5% CO_2_. All the cell cultures were tested negative for mycoplasma contamination.

### Single-cell dissociation

The single-cell suspensions of SVZ and DG were prepared as described previously with modifications [24]. We used eight male and eight female mice (two male and two female mice at each age) in two independent experiments, with each dataset (dataset A and dataset B) being sampled on the same day.

### 10× genomics chromium

After dissociation, cell suspensions in a concentration of 600–1000 cell/μL in ice-cold PBS with 0.04% bovine serum albumin were loaded into a Chromium Single Cell 3ʹ Chip (10× Genomics) and processed following the manufacturer’s instructions. Sequencing was carried out using different versions of 10× Genomics Chromium Single Cell Kit (dataset A by Version 3.0 while dataset B by Version 3.1).

### Sequencing data processing and quality control

scRNA-seq data were aligned to the GRCm38 (mm10) mouse reference genome individually using Cell Ranger 3.0 pipelin. Basic processing, filtering, classification, and visualization of the aggregated scRNA-seq data were analyzed with the Seurat package (v.2.3) in R (v.3.3.4). Cell types were identified based on the expression of canonical marker genes for each cluster. Uniform manifold approximation and projection (UMAP) was then utilized for two-dimensional visualization of the cells in SVZ and DG separately.

### Pathway, differential expression, cell trajectory, and cell cycle analysis

Pathway analysis, DEG analysis, cell trajectory analysis, and cell cycle analysis were performed as described in Supplementary Methods.

### Intercellular communication computation

Cell-cell communication analysis, curation of known ligand-receptor interactions (Table S1), and the calculation of intercellular communication intensity were performed as described in Supplementary Methods.

### Western blotting

Cells or tissues comprising the lateral ventricle wall and hippocampi were lysed in ice-cold RIPA buffer containing protease inhibitor cocktail, 1 mM PMSF, 1 mM NaF, and 1 mM EDTA. After centrifugation, the protein extractions were separated by SDS-PAGE and followed by incubation with primary antibodies for 1 h at room temperature and 4°C overnight. Then, horseradish peroxidase-conjugated secondary antibodies were incubated for 2 h at room temperature. The bands were visualized by Chemiluminescence.

### In situ hybridization

*In situ* hybridization (ISH) was carried out as previously described [30]. 387 bp mouse cDNA of CPE was used as a template for *ISH* probes. Primers used were as follows: Fd: GGAGCGGAGGCTGTAAGTTT; Rs: ACCTCCCTGTCGCAAGAATG.

### Immunohistochemistry and confocal microscopy image analysis

Immunostaining for cultured cells or brain slices were performed as described previously [30]. See Supplementary Methods for details.

### BrdU administration

For analysis of cell proliferation in the brain, mice were given three injections of BrdU (50 mg/kg body weight, i.p.) within 24 h, and analyzed 24 h after the last injection.

### Recombinant lentivirus production

For CPE overexpression, CPE cDNA was cloned into modified SBP-Flag-tagged pWPXL lentiviral vector. Lentiviral particles were prepared as described previously [31].

### Isolation, culture, and lentiviral infection of primary NSCs

Primary NSCs were isolated from the SVZ of 8-week-old WT mice based on published methods [32]. For a typical infection of each 24-well plate containing primary NSCs, we added 1 μL of concentrated lentiviral particles to 500 μL of Neurobasal-A medium. The NSCs were used for immunostaining and western blotting experiments 48 h later.

### Osmotic pump grafting

Infusion of mice was performed using Alzet mini-osmotic pump (Model 1007D). The cannula was implanted stereotaxically in the left lateral ventricle (antero-posterior, 0.5 mm; lateral, 1.3 mm; depth, 2.9 mm relative to bregma and the surface of the brain).

### Enzymatic activity assay for CPE-WT and CPE-E342Q

Recombinant human CPE-WT and CPE-E342Q protein were produced in HEK293T cells. Enzymatic activity of CPE-WT and CPE-E342Q were tested as described previously [33].

### In vitro proBDNF cleavage

Protease cleavage of proBDNF *in vitro* was performed as described [34]. The conversion ratio from proBDNF to mBDNF were determined by Western blotting.

### Statistical analysis

For all experiments, at least three biological or experimental replicates were analyzed. Sample size for each experiment is indicated in the corresponding figure legend. All data are presented as mean ± SEM unless stated otherwise. Statistical significance of data were analyzed using two-tailed unpaired Student’s *t* test. Data from multiple groups were analyzed with one-way analysis of variance (ANOVA) followed by Dunnett’s multiple comparisons test. Significance was set at *P* < 0.05, *P* < 0.01, and *P* < 0.001. Specific *P* values are indicated in the figures.

### Data availability

The scRNA-seq data have been deposited in the Genome Sequence Archive [35] in BIG Data Center, Beijing Institute of Genomics, Chinese Academy of Sciences, China National Center for Bioinformation, with accession number GSA: CRA003819 (project PRJCA004342).

## Discussion

**Figure 7.**
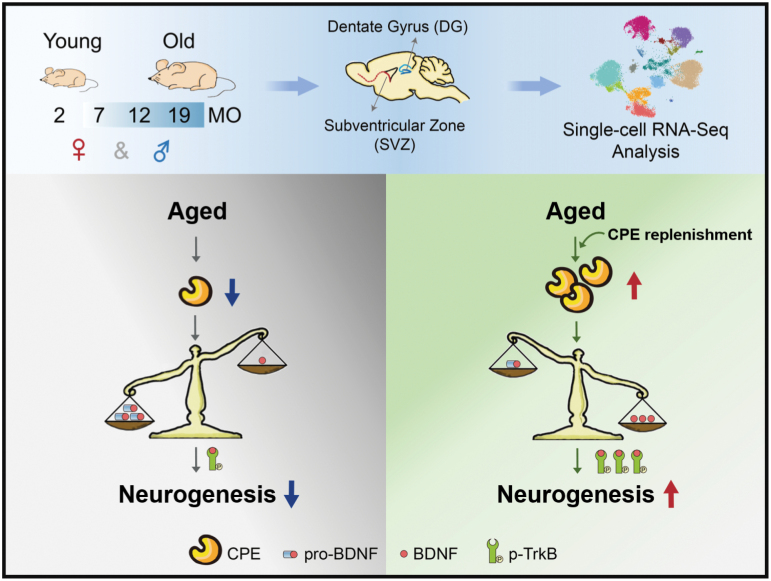
**Graphical summary.** Comprehensive single-cell transcriptomic analyses of SVZ and DG identifies CPE as a target for stimulating adult neurogenesis in aging brain. A single-cell transcriptome examination of both SVZ and DG regions in the brains from female and male mice at four different ages (2-, 7-, 12-, and 19-months of age, representing adulthood onset, middle-aged, reproductive senescence, and senescence phase, respectively) was performed (upper panel). Aging caused a deficiency of BDNF-TrkB signal cascade in old NSCs due to decreased mature form of BDNF and its receptor TrkB, thus limiting the neurogenesis in old neurogenic niches (lower-left panel). CPE, a carboxypeptidase highly enriched in NSCs and significantly downregulated with aging, played a crucial role in mature BDNF generation. Restoring the expression of CPE in aged mice rebuilt BDNF generation and neurogenesis (lower-right panel).

In the present study, our scRNA-seq analysis provided an unprecedented opportunity to compare the neurogenesis directly and systematically at single-cell level in the two discrete neurogenic regions from both sexes across the mouse lifespan, which not only highlight the consideration of region- and sex-differences in applying neurogenesis for regenerative medicine and precision medicine, but also allow us to give a detailed view of cell-cell communications in these niches based on receptor-ligand interactions. Intriguingly, receptor-ligand analyses demonstrated a deficiency of BDNF-TrkB signaling cascade in old NSCs, and identified CPE as a potent drug target to rebuild the BDNF generation and neurogenesis in aged brain niches (Fig. 7).

Our findings that intracerebral infusion of the native or WT CPE, but not the heat inactivated or enzymatic inactive mutant CPE-E342Q, significantly boost adult neurogenesis, suggesting that this action of CPE is dependent on its enzymatic activity. However, how exactly infusion of CPE leads to increased neurogenesis is unclear. CPE has been reported to function intracellularly as an exopeptidase and act as a sorting receptor for proBDNF [42, 45]. During infusion, a portion of CPE might be internalized into NSCs and contributes positively to the convertases-mediated proBDNF processing in an enzymatic activity-dependent manner. However, we cannot rule out the possibility that the neurotrophic effect of CPE itself [48, 56, 57], especially those are not taken up into cells in the neurogenic regions, as well as the neuroprotection effect mediated by the binding of extracellular CPE to a receptor (HTR1E) [49], might also be involved. Therefore, it is possible that both intracellular and extracellular mechanisms contribute to the enhanced neurogenesis upon CPE infusion, with the contribution extent undefined. Moreover, our transcriptomic data showed that astrocytes are the primary niche cells that express *Cpe*. However, we found that the CPE expression is much higher in NSCs and their progeny compared to the niche astrocytes, suggesting that CPE is primarily induced in NSCs. Giving that CPE is a secreted protein, we speculate that the neurogenesis boosting effect of CPE is achieved, to some extent, by the reciprocal regulation between adult NSCs and niche cells. The exact underlying mechanism deserves further exploration.

The mechanism of how the imbalanced BDNF/proBDNF occurred in the SVZ in the current study deserves deeper thinking. Our data have demonstrated that CPE replenishment upregulates mBDNF levels in an enzymatic activity-dependent manner. Given that CPE works best at acidic pH, and processing of proBDNF occurs in regulated secretory vesicles at an acidic pH, we speculate that CPE might be involved in the proBDNF processing intracellularly. We then demonstrate that PC2 co-localizes with CPE in NSCs and cleaves proBDNF, while CPE promotes its cleaving efficiency. The significant accumulation in full-length proBDNF in the SVZ of CPE knockdown NSCs suggests that the proBDNF processing is somewhat impaired. Therefore, this process holds some functional importance, even if currently undefined.

Dysregulated BDNF-TrkB signaling is reported to be associated with Alzheimer’s disease and other neurodegenerative disorders [58, 59]. However, due to the short half-life of mBDNF in blood and its inability to across the blood-brain barrier [58], delivering mBDNF as a neuroprotective agent is impracticable. Manipulating the involved proteases represents candidate targets to regulate the balance of BDNF isoforms. Our findings that replenishment of CPE expression promotes mBDNF generation and boosts adult neurogenesis suggest that manipulating CPE-controlled BDNF-TrkB signaling pathways may be a promising approach for facilitating endogenous adult NSC expansion for the treatment of certain disorders in regions associated with constitutive neurogenesis.

## Research limitations

One of the limitations of our study is the relatively small number of mature neurons obtained for the scRNA-seq analysis, which might be due to the high sensitivity of mature neurons in adult/aged brains to tissue dissociation procedure. In addition, the conclusions that CPE promotes the cleaving efficiency of PC2 are based on Western blot analysis, which does not assess the kinetics of proBDNF processing *in vivo*, and the detection of intermediate forms may be below the sensitivity of our immunoblots. Future mechanistic insights are needed to answer the following questions. For instance, is the proBDNF processing by PC2 and CPE a one- or two-step process? If it is the latter, what is the order of these cleavage events and the exact cleavage site?

## Supplementary Material

lnad015_suppl_Supplementary_Materials

lnad015_suppl_Supplementary_Table_S1

lnad015_suppl_Supplementary_Table_S2

lnad015_suppl_Supplementary_Table_S3

lnad015_suppl_Supplementary_Table_S4

lnad015_suppl_Supplementary_Table_S5
