# Supplementary material for: Restoring carboxypeptidase E rescues BDNF maturation and neurogenesis in aged brains
Source: Life Med. 2023 Apr 11;2(2):lnad015. doi: 10.1093/lifemedi/lnad015 (PMC11749474; doi:10.1093/lifemedi/lnad015)

**Supplementary Materials**

**Restoring Carboxypeptidase E Rescues BDNF Maturation and Neurogenesis in Aged Brains**

**Supplementary Methods**

*1. Antibodies*

The following primary antibodies were used for Western blotting: mouse anti-actin (1:50,000, Proteintech, 66009-1-lg), rabbit anti-BDNF (1:3000, Genetex, GTX134514), rabbit anti-proBDNF (1:1000, ThermoFisher Scientific, PA5-77533), Rabbit anti-TrkB (1:1000, Proteintech, 13129-1-AP), rabbit anti-CPE (1:1000, Proteintech, 10069-T40).

The following primary antibodies were used for immunohistochemistry: chicken anti-nestin (1:100, Abcam, ab134017), rabbit anti-SOX2 (1:400, Abcam, ab97959), rabbit anti-Ki67 (1:300, Abcam, ab15580), rabbit anti-doublecortin (1:200, Abcam, ab18723), rat anti-BrdU (1:500, Abcam, ab6326), goat anti-EGFR (1:100, Novus Biologicals, AF1280), mouse anti-MCM2 (1:200, Novus Biological, NBP2-37566), rabbit anti-BDNF (1:1000, Abcam, ab108319), rabbit anti-UBE2C (1:200, Proteintech, 12134-2-AP), rabbit anti-CPE (1:100, Proteintech, 13710-1-AP), rabbit anti-TrkB (1:100, Proteintech, 13129-1-AP), rat anti-THBS4 (1:100, Novus Biologicals, MAB7860), goat anti-CPE (1:200, R&D Systems, AF3587), goat anti-TrkB (1:100, R&D Systems, AF1494), mouse anti-HMGB2 (1:1000, Novus Biological, H00003148-M05), rabbit anti-UNG (1:100, Novus Biological, Nb600-1031), mouse anti-PSA-NCAM (1:100, Millipore, MAB5324), rabbit anti-phospho-TrkB (Tyr515) (1:200, Abcam, ab131483), rabbit anti-proBDNF (1:1000, Thermo Fisher Scientific, PA5-77533), mouse anti-GFAP (1:400, Chemicon, MAB360), rabbit anti-GFAP (1:5000, Dako, Z0334).

*2. Single cell dissociation*

Briefly, after each mouse was decapitated, the SVZ and DG regions were simultaneously micro-dissected and submerged in fresh ice-cold and oxygenated Hibernate-A medium (GIBCO). Then, the dissected tissues were digested with 20 U/mL Papain (Worthington) and 250 U/mL DNase I (Roche) in the Hibernate medium for 30 min at 37°C. Cold Hibernate medium containing 10% FBS was added to quench the enzymatic reaction. The tissue pieces were dissociated into single cells by gentle trituration followed by serial filtrations through pre-

wetted 40-μm (Falcon) and 30-μm cell strainers. Myelin debris and erythrocyte removal step was omitted to prevent any bias in the recovered cell yields. All cell sedimentations were performed at 130 g for 5 min at 4°C. Oxygenated Hibernate-A medium was used in all steps, and cells were kept on ice except the enzymatic digestion stage.

*3. Pathway, differential expression cell trajectory and cell cycle analysis*

Neurogenic cells were extracted using the SubsetData function in Seurat. Sub-clusters were further identified with a clustering resolution set to 0.4. Pseudotime analyses were performed using the Monocle package, as described in the tutorials (http://cole-trapnell-lab.github.io/monocle-release/).

Gene expression matrix of all cells in the SVZ or DG were generated by Seurat after log normalization. The gene expression level in a cell was quantified as the transcripts per million (TPM). The coordinate information and cluster information of each cell were extracted using the data@dr$umap@cell.embeddings and data@ident parameters in Seurat package. Differential expression analysis was performed by Limma package in R 3.4.2. For each cluster, cells from the 2 and 19 MO mice were selected for aging-caused differential expressed genes (DEG) analysis. For analysis of DEGs between brain regions, we used a *P*-value less than 0.05. Heatmaps were generated using the pheatmap function in R 3.5.2.

Analysis of cell cycle status was performed with R package scran.

*4. Intercellular communication computation*

Analysis of cellular communication is based on the ligand-receptor interactions. Single cells of SVZ were investigated using the CellPhoneDB software to determine interaction networks. Interaction pairs with *P*-value < 0.05 were remained. The numbers of potential ligand-receptor pairs between cell groups were shown by heatmap.

Curation of known ligand-receptor interactions: Computationally inferred ligand–receptor pairs were downloaded from the DLRP and CellPhoneDB databases. Gene lists of ligands/receptors involved in interacting pairs were manually curated using cell-cell interaction database, Genecard and Uniprot to correct or remove genes that were misclassified. To predict cell-cell interactions, the ligand-receptor pair dataset was further filtered for genes detected in our scRNA-seq transcriptional dataset, which results in a final curated set of 250 receptors and 196 ligands (expressed in at least five cells), leading to 449 interacting ligand-receptor pairs.

Specifically, 69 receptors were expressed in NSCs, which formed 223 pairs with 103 ligands, while 54 ligands were expressed by NSCs, which formed 142 pairs with 79 receptors.

Intercellular communication score computation: Firstly, since mutual information has been successfully applied to transcriptional network inference, we measured correlation between the particular receptor and ligand comprising an interacting pair across total cells in SVZ, using normalized mutual information ($nMI$) as below,

$$nMI = I(X;Y) = \sum_{x \in X} \sum_{y \in Y} p(x,y) \log \frac{p(x,y)}{p(x)p(y)} \qquad \text{(Equation 1)},$$

where $x$ is the TPM of a given receptor, $y$ is the TPM of the corresponding ligand involved in a pair in each cell of SVZ, $X$ is the set of TPMs of this receptor, and $Y$ is the set of TPMs of the corresponding ligand in total cells of SVZ.

Secondly, we quantified the intensity of each ligand-receptor interaction between NSCs and the partner cells (NSCs and their niches) by computing the score based on their expression profiles. For a particular ligand-receptor interaction, the interaction intensity was computed using the following formula,

$$score = nMI \cdot \frac{\sum_{i=1}^{n} TPM_i^q}{n} \cdot TPM^p \qquad \text{(Equation 2)},$$

where $TPM^q$ is the TPM of a receptor or ligand in the NSCs, $TPM^p$ is the expression level of the corresponding communication molecule by the specific single partner cell in the SVZ, and n is the total number of receptors/ligands in the NSCs.

A composite score, $Score,$ was used to measure the communication intensity between NSCs and each partner cell, which was defined by summing up the intensity of all the possible ligand-receptor interactions, i.e.,

$$Score = \sum_{i=1}^{N} score_i \qquad \text{(Equation 3)},$$

where $N$ is the total number of ligand-receptor pairs between NSCs and the partner cells.

*5. Immunohistochemistry and confocal microscopy image analysis*

Immunostaining for cultured cells or brain slices were performed according to the following procedure. Free-floating sections were removed from the cryoprotectant and exhaustively washed in PBS for 60 min. Sections were then blocked in PBS with 5% (v/v) normal donkey/goat serum, 1% (w/v) BSA and 0.3% (v/v) Triton X-100 for 1 h at room

temperature. After blocking, sections were incubated with the respective primary antibody at 4ºC overnight. On the next day, appropriate secondary antibodies (all 1:1000, ThermoFisher) were applied for 2 h at room temperature. Thereafter, sections were washed 3 × 15 min in TBST (pH 7.6), followed by counterstaining with the fluorescent nuclear dye Hoechst 33342, then mounted with mounting medium and kept at 4ºC until further microscopic analysis.

For quantification of BrdU-positive cells in the adult brain, free-floating sections were treated with 2N HCl at 37ºC for 30 min, and neutralized by washing with 0.1 M sodium borate buffer (pH 8.5) for 10 min at room temperature. Sections were blocked in 5% donkey serum in Tris-buffered saline plus 0.5% Triton X-100 for 1 h, and subsequently incubated with rat anti-BrdU antibody (1:500) overnight at 4ºC, followed by Alexa Fluor donkey anti-rat 488 nm antibody (1:1000) for 2 h.

For immunolabelled mouse brain sections, images were acquired using a Nikon A1R Eclipse Ti confocal microscope (Nikon, Japan). For laser scanning confocal microscopy, z-stacks with optical sections of 1.5 μm were recorded. ImageJ was used for image processing and quantification. Images of the entire V-SVZ were taken for both V-SVZ of each section. Stereology was performed by the analysis of 6 coronal sections per animal. $BrdU^+$ and $BrdU^+DCX^+$ cells within the SVZ were counted using every $6^{th}$ section (180 μm apart). The SVZ was analyzed comprising the region between 1.18 anterior to 0.02 mm posterior to the bregma. The number of $BrdU^+$ and $BrdU^+DCX^+$ cells located in the cell dense region around the ventricle were manually counted in entire coronal sections while directly visualizing each section under the microscope with the 20 × objective. The number of counted cells in each section was normalized to the area of the SVZ, then multiplied by six. The total positive cell number in the analyzed region per animal was calculated by summarizing the total cell number per 180 μm region.

For APOE fluorescence quantification, the entire SVZ area in both brain hemispheres was imaged using the 40 × objective. ImageJ was used for image analysis. The APOE fluorescence co-localizing with HMGB2 was quantified as follow: the channel for HMGB2 was used to generate a mask by setting intensity threshhold to outline the positive areas. The same threshold values were used for all images across all conditions in each individual experiment. The cell masks were then used to determine the APOE fluorescence levels by mean intensity per masked pixel. The mean intensity APOE values were then normalized for each independent experiment

by dividing them by the mean intensity of the 2 MO samples and the mean of these normalized values was used for plotting.

For TrkB, p-TrkB, proBDNF, BDNF and CPE fluorescence analysis, the SVZ area in both brain hemispheres was imaged using the 40 × objective. ImageJ was used to measure the mean fluorescence intensity. The fluorescence analysis in the SVZ were performed on a rectangular area of 250 × 100 μm box drawn in the dorsal domain of the SVZ encompassing the protein expression domain. The area of the box was kept the same for all samples analyzed. These areas were then used to determine the mean fluorescence intensity of the target protein, which was then normalized to the number of cells contained in the ROIs for each independent experiment. At least three brain sections at different rostral/caudal levels from each animal were randomly selected, analyzed and averaged, from at least three animals each group.

For cell culture assays, images used for quantifications were acquired with a Nikon A1R Eclipse Ti confocal microscope with 20 × objective. The immunoreactivity measurements were performed by using ImageJ and the mean fluorescence intensity was normalized to the nuclear fluorescence.

*6. In vivo loss of function via lentivirus stereotaxic injections to SVZ*

For ICV delivery, equal volume and similar titer of lentivirus (LV-shRNA-CPE-CDS-1, targeting sequence : CCGGGGATTACTGGCGATTGCTTGCCTCGAGGCAAGCAATCGCCAGTAATCCTTTTTG; or LV-shRNA-CPE-CDS-2, targeting sequence: CCGGGGTTGTGTGCAGTTGATATTTCTCGAGAAATATCAACTGCACACAACCTTTTTG, or LV-shRNA-NC, CCGGTTCTCCGAACGTGTCACGTTCTCGAGAACGTGACACGTTCGGAGAATTTTTTG) in PBS were injected (at 0.2 μL per minute) into the lateral ventricles using a microsyringe (Hamilton) under the guidance of a stereotaxic instrument (RWD Life Science). The needle was left in place for an additional 5 min after injection to prevent possible leakage. The injection coordinates for intracerebroventricular injection to the left lateral ventricle were: antero-posterior = 0.5 mm relative to bregma; lateral = 1.3 mm to the midline; and depth = 2.9 mm down from the surface of the skull. Mice were allowed to recover for 7 days, followed by BrdU intraperitoneal injections (50 mg/kg body weight) and analyzed at 24 h post-injection.

*7. ANA12 administration*

Systemic administration of 0.5 mg/kg of ANA12 (Selleck, Cat#S7745) was performed by intraperitoneal injection every other day for 14 days.

**Figure S1. Construction, classification and profiling of single cell atlases. Related to Fig. 1. (A)** The library building process of single cell transcriptome sequencing. SVZ and DG cells were collected from eight mice at 2, 7,12 and 19 MO (one female and one male at each age) for Dataset A and Dataset B, respectively. **(B)** Marker genes used for the identification of astrocytes and NSCs in the SVZ (Dataset A). The color key indicates expression levels. The blue circle in the middle and bottom panels highlights the expression of NSC marker genes and the absence of mature astrocyte markers in NSCs. **(C)** UMAP visualization colored by the expression of selected marker genes of the SVZ Dataset A. The color key indicates expression levels. **(D)** UMAP visualization colored by the expression of selected marker genes of the DG Dataset A. The color key indicates expression levels. The blue circle in the top panels highlights the absence of mature astrocyte markers and the expression of NSC marker genes in NSCs. **(E and F)** Heatmap showing the scaled expression of the top markers of each cell type in the SVZ (E) and DG (F) (Dataset A). **(G)** UMAP plot of cells from Dataset B showing 15 clusters in the SVZ (left panel, eight samples, 33, 068 cells) and 18 clusters in the DG (right panel, eight samples, 66, 720 cells). Cells are colored by different cell types. **(H)** Statistics of the cell number and totally expressed genes (over five cells) of major cell types in the SVZ and DG from combined analysis of Dataset A and Dataset B. Data are represented as mean ± S.E.M. **(I)** Marker genes used for the identification of astrocytes and NSCs in the SVZ (Dataset B). The color key indicates expression levels. The blue circle highlights the expression of *Slc1a3*, *Fabp7*, NSC marker genes (*Igfbp5*, *Nr2e1*, *Lrig1* and *Thbs4*) and the absence of mature astrocyte markers (*Grin2c* and *Aqp4*) in NSCs. **(J)** UMAP clustering as in Figure 1A, 1B and supplementary Fig. 1G, but colored by age and sex of SVZ and DG (Top four panels, Dataset A; Bottom four panels, Dataset B).

**Figure S2. Stem cell related DEGs in the SVZ and DG and profiling of sex- and aging-caused DEGs. Related to Fig. 1. (A, B and C)** Heatmap plots for DEGs of NSCs (A), TAPs/IPCs (B) and NBs (C) between SVZ and DG regions (based on Dataset A). **(D)** Representative images of APOE expression in the SVZ and DG of mice at 2, 8, 12 and 18 MO. Scale bars, 10 μm. (**E**) Venn diagram of DEGs of female and male mice in the SVZ region between 2 MO and 19 MO (combined analysis of Dataset A and B). 19FM up, upregulated

DEGs of 19 MO female mice. 19MM up, upregulated DEGs of 19 MO male mice. 19MM down, downregulated DEGs of 19 MO male mice. 19FM down, downregulated DEGs of 19 MO female mice. **(F)** The volcano plot for the DEGs between 2 MO and 19 MO in SVZ of female mice and male mice. Upregulated genes in 19 MO are colored in red, while downregulated colored in blue. Top leading significant DEGs are labelled by gene name (combined analysis of Dataset A and B). **(G)** Boxplot for the expression of *Prdx1* for the cells that do express the gene at each age group in female and male mice (combined analysis of Dataset A and B). **(H)** Representative images of PRDX1 expression in the SVZ of female and male mice at 2 and 18 MO. Scale bar, 20 µm. Quantification of relative fluorescence intensity of PRDX1 expression was shown on the right panel. Data are represented as mean ± S.E.M. *n* = three mice per age.

**Figure S3. Profiling of cell cycle active and inactive TAPs. Related to Fig. 2. (A)** UMAP visualization of NSCs at different stages colored by ages (Dataset A). **(B)** UMAP plot of the NSCs, TAPs and NBs in the SVZ based on dataset B. **(C)** The representative GO terms of DEGs between cell cycle active and inactive TAPs (Dataset A). **(D)** Volcano plot for the DEGs between cell cycle active and inactive TAPs in the SVZ (Dataset A). **(E)** Density plot for expression of cell cycle related genes in cell cycle active (red) and inactive (blue) cells (Dataset A). **(F)** UMAP visualization of supplementary Fig. 2B colored by the expression of representative stem cell marker genes (Dataset B). Different color indicates different markers used to identify each cell type. Green: NSCs; blue: cell cycle inactive TAPs; orange: cell cycle active TAPs; red: NBs. **(G)** Pseudotime trajectory of NSCs in the SVZ colored by the expression of *Egfr*, *Ung*, *Ube2c*, *Dcx* and *Stmn2* (Dataset A). **(H)** UMAP plot of the IPCs in the DG (combined analysis of Dataset A and B). (**I**) UMAP visualization of supplementary Fig. 2I colored by the expression of cell cycle-related genes (combined analysis of Dataset A and B). (**J**) Bar plot for statistics of the cell counts of total TAPs, cell cycle active and inactive TAPs in the SVZ of mice at different ages from all the replicates (combined analysis of Dataset A and B). Data are represented as mean ± S.E.M. *n* = four mice at each age. *P* values are indicated (one-way ANOVA). (**K**) Immunostaining for BrdU, Ki67 and HMGB2 in the DG of 2 MO WT mice. Scale bar, 20 µm.

**Figure S4. Cell-to-cell communication analysis in the SVZ and DG. Related to Fig. 3. (A)** Heatmap showing numbers of potential ligand-receptor pairs between cell groups in the SVZ (Dataset A) predicted by CellPhoneDB. **(B)** UMAP plot showing global communication scores, combining the intensity of all the individual ligand/receptor interactions between ligands expressed by each cell in the SVZ and corresponding receptors expressed by NSCs (left), as well as between ligands expressed by NSCs and corresponding receptors expressed by each cell in the SVZ (right). **(C)** Expression plot of ligands involved in ligand-receptor interactions over the UMAP map to assess ligand-receptor interaction prevalence. **(D)** Volcano plot of DEGs between NSC-$R_H$ and NSC-$R_L$ in the SVZ (Dataset A). **(E)** Analysis about heterogeneity of NSCs in the DG based on the receptor expressions (Dataset A). Sub-clustering analysis using unsupervised hierarchical clustering of NSCs revealed two subclusters (left). UMAP plot of NSCs showing the expression of receptors involved in ligand-receptor interactions (right). **(F)** Analysis about heterogeneity of NSCs in the SVZ based on the receptor expressions (Dataset B). Subclustering analysis using unsupervised hierarchical clustering of NSCs revealed two subclusters (Left). UMAP plot of NSCs showing the expression of receptors involved in ligand-receptor interactions (Right). **(G)** UMAP visualization of supplementary Fig. 4F colored by the expression of genes involved in activation of qNSCs (*Ascl1*, *Egfr* and *Cd9*). NSC-$R_H$ show higher expression of activation markers. **(H)** Analysis about heterogeneity of NSCs in the DG based on the receptor expressions (Dataset B). **(I)** Bar plot for statistics of the cell numbers of NSC-$R_H$ and NSC-$R_L$ in the SVZ at 2, 7, 12 and 19 MO. Data are represented as mean ± S.E.M. $n$ = four mice at each age (combined analysis of Dataset A and Dataset B). $P$ values are indicated (one-way ANOVA). **(J)** Immunofluorescence images of classic markers THBS4, SOX2 and GFAP for NSCs, which were dominantly expressed in qNSCs (Top panel). Immunofluorescence images of known activation marker EGFR, as well as Nestin, in THBS4$^+$ qNSCs. Yellow arrowhead represents THBS4$^+$EGFR$^+$Nestin$^+$ primed qNSCs (Lower panel). Scale bars, 10 μm. **(K)** Volcano plot of differentially receptor genes between NSC-$R_H$ and NSC-$R_L$ in the DG (Left, Dataset A, Right, Dataset B) and SVZ (Middle, Dataset B). **(L)** Representative images of the SVZ stained with TrkB, THBS4 and EGFR in 2-MO mice. Scale bar, 10 μm. White arrowheads indicate THBS4$^+$TrkB$^+$EGFR$^{low}$ cells. **(M)** Aging-related changes in intercellular communication in the DG. Ligands and receptors that interact are lined by a chain of arrows, the color of which means the nMI scores of the given ligand and receptor. The exact niche cell types that interact with NSCs for each ligand or receptor were shown on the right.

**Figure S5. *In vivo* characterization of TrkB, BDNF and CPE expression in the SVZ. Related to Fig. 4. (A)** Characterization of TrkB-positive NSCs (GFAP$^{+}$), TAPs (HMGB2$^{+}$) and NBs (DCX$^{+}$) in the SVZ of 2 MO mice. Ho, Hoechst. Scale bars, 20 μm. **(B)** Characterization of p-TrkB-positive NSCs (SOX2$^{+}$GFAP$^{+}$), TAPs (HMGB2$^{+}$) and NBs (PSA-NCAM$^{+}$) in the SVZ of 2 MO mice. Scale bars, 20 μm. **(C)** Boxplot for the expression of *Ntrk2* in the NSCs of the SVZ that do express the gene at each age group. **(D)** Representative images of BDNF and NT-4 expression in the NSCs of the SVZ from 2 MO female mice. Scale bars, 20 μm. **(E)** Characterization of BDNF-positive NSCs (Nestin$^{+}$GFAP$^{+}$), TAPs (HMGB2$^{+}$) and NBs (PSA-NCAM$^{+}$) in the SVZ of 2 MO mice. Scale bars, 20 μm. **(F)** The volcano plot for the DEGs of NSCs between 2 MO and 19 MO in the SVZ (Dataset A). **(G)** Boxplot for the expression of *Cpe* in the NSCs of the SVZ that do express the gene at each age group.

**Figure S6. Restoring CPE promotes adult neurogenesis in the SVZ. Related to Fig. 5. (A)** Normalized CPE fluorescence intensity in the ipsilateral and contralateral SVZ from 18 MO mice one week after PBS or CPE infusion, respectively. Data are represented as mean ± S.E.M. *n* = three mice per group. *P* values are indicated (one-way ANOVA). ipsi., ipsilateral; contra., side contralateral to CPE injection. **(B)** (Left) Representative images of GFAP and Iba1 expression in the ipsilateral and contralateral SVZ from 18 MO mice one week after CPE infusion. Ho, Hoechst. Scale bars, 10 μm. (Right) Quantification of GFAP$^{+}$ and Iba1$^{+}$ cells in the ipsilateral and contralateral SVZ from 18 MO mice one week after CPE infusion, respectively. Data are represented as mean ± S.E.M. *n* = three mice per group. *P* values are indicated (two-tailed t-test). **(C and D)** Normalized p-TrkB, proBDNF, BDNF (C) and p-ERK (D) fluorescence intensity in the ipsilateral and contralateral SVZ from 18 MO mice one week after CPE infusion, respectively. Data are represented as mean ± S.E.M. *n* = three mice per group. *P* values are indicated (two-tailed t-test). **(E)** Left, Representative images of BrdU and DCX double-labelled newly generated neurons in the SVZ from 12 MO mice and 9 MO mice one week after PBS or CPE infusion, respectively. Quantification of BrdU$^{+}$ and BrdU+DCX$^{+}$ cells in the SVZ was shown on the right. Data are represented as mean ± S.E.M. *n* = three mice per group. *P* values are indicated (one-way ANOVA). **(F)** Representative images of THBS4, Ki67, DCX and PSA-NCAM expression in the ipsilateral and contralateral side of SVZ from 9 MO mice one week after CPE infusion. Scale bars, 20 μm. **(G)** Representative images of

proBDNF and BDNF expression (Left) and normalized fluorescence intensity (Right) in the ipsilateral and contralateral SVZ from 9 MO mice one week after CPE infusion. Scale bars, 10 μm. Data are represented as mean ± S.E.M. *n* = three mice per group. *P* values are indicated (two-tailed t-test). **(H)** Western blotting analyses (Left) and relative quantification expression level (Right) of proteins extracted from the mixed tissues of SVZ from three mice after PBS or CPE infusion. β-actin is used as a loading control. *P* values are indicated (two-tailed *t*-test). **(I)** Normalized proBDNF and BDNF fluorescence intensity in the SVZ of mice with grafted LV-NC or LV-CPE. Data are represented as mean ± S.E.M. *n* = three mice each group. *P* values are indicated (two-tailed *t*-test). **(J)** Normalized CPE, proBDNF and BDNF fluorescence intensity in the SVZ of mice injected with lentiviruses expressing shNC or shCPE_2. Data are represented as mean ± S.E.M. Each dot represents mean normalized protein fluorescence in NSCs in three sections from one mouse. *n* = three mice each group. *P* values are indicated (two-tailed *t*-test). **(K)** Relative quantification of Western blotting analysis of protein levels in the SVZ of mice injected with lentiviruses expressing shNC or shCPE_2. Data is represented as the mean protein intensity normalized to β-actin ± S.E.M. from three independent mice each group. *P* values are indicated (two-tailed t-test).

**Figure S7. Characterization of *in vivo* expression and *in vitro* cleavage activity of different peptide precursor processing enzymes. Related to Fig. 6. (A)** Representative immunofluorescence images of primary NSCs stained with Nestin and SOX2 in adherent cultures. Ho, Hoechst. Scale bar, 20 μm. **(B)** Co-staining of furin, MMP-9, plasminogen or tPA expression with NSC markers in the SVZ. Scale bars, 10 μm. **(C)** Western blotting analyses of proBDNF and mBDNF by incubating commercial recombinant proBDNF with furin, PC1/3, PC2, MMP-9, plasmin with or without CPE at 37°C *in vitro*, respectively. **(D)** Western blotting analyses of the purified CPE-WT and CPE-E342Q protein from 293T cells. **(E)** The specific enzymatic activity of recombinant CPE-WT and CPE-E342Q protein by incubating with Benzoyl-Ala-Arg-OH substrate at 37°C for 20 min, recording fluorescence and calculating specific activity (nmol/min/μg). **(F)** Normalized proBDNF and BDNF fluorescence intensity in the SVZ of native CPE-infused mice with or without GEMSA treatment. Data are represented as mean ± S.E.M. n = three mice each group. *P* values are indicated (two-tailed *t*-test). **(G)** Normalized proBDNF and BDNF fluorescence intensity in the SVZ of mice with grafted LV-CPE-WT or LV-CPE-E342Q. Data are represented as mean ± S.E.M. *n* = three mice each group. *P* values are indicated (two-tailed t-test).

**Supplemental Table caption**

Table S1. Manually curated list of ligand/receptor genes, interacting pairs and the specifical pairs of NSCs.

Table S2. Top markers in each cell type in SVZ and DG (Dataset A and B).

Table S3. Statistics of DEGs between SVZ and DG of each major cell type.

Table S4. DEGs and enriched GO terms of stem cell types (NSCs, TAPs/IPCs, NBs) in the SVZ and DG.

Table S5. GO terms of each aging-caused DEGs in each major cell type of SVZ and DG.

## Supplementary Figure 1

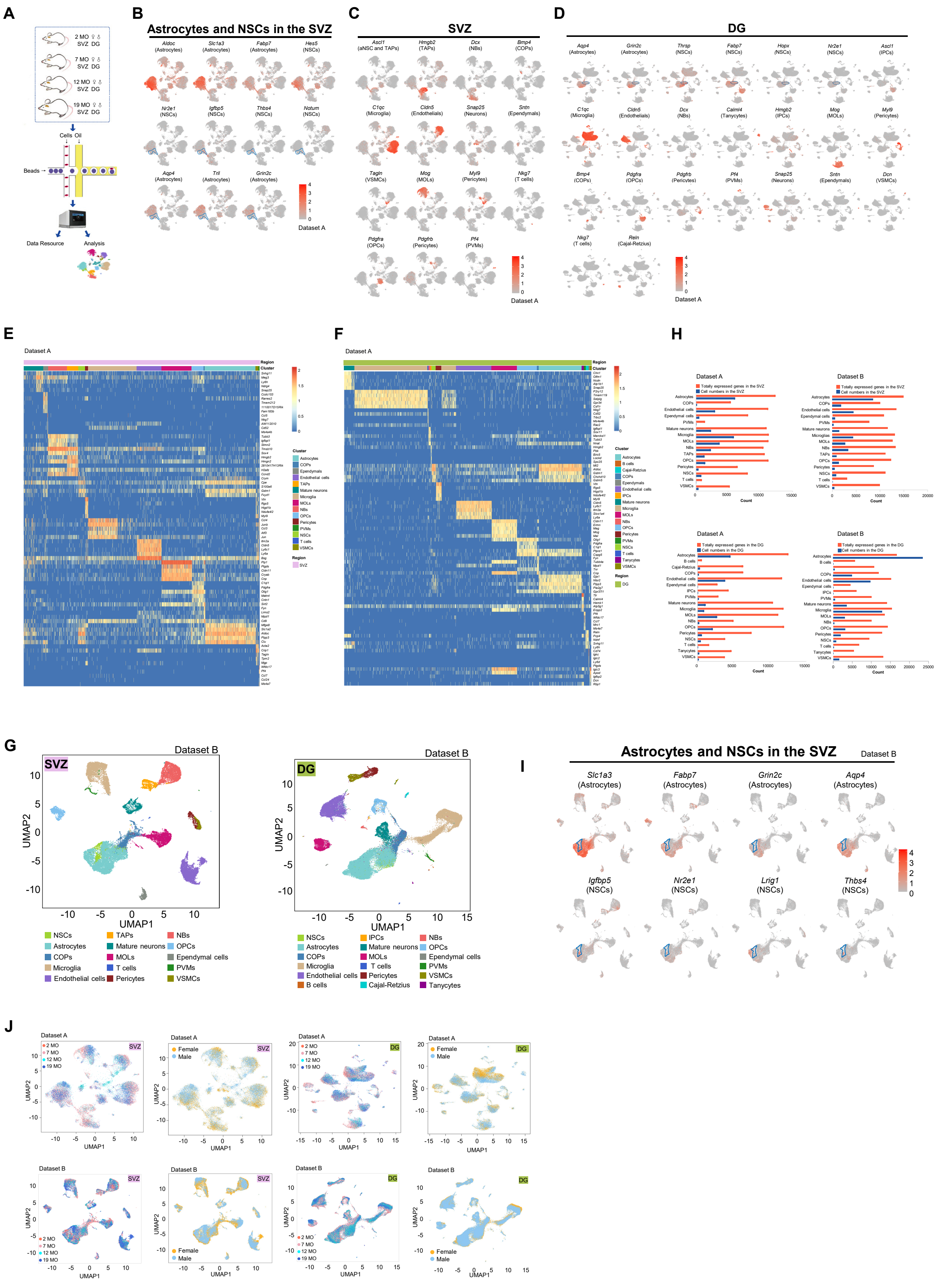

**Supplementary Figure 2**

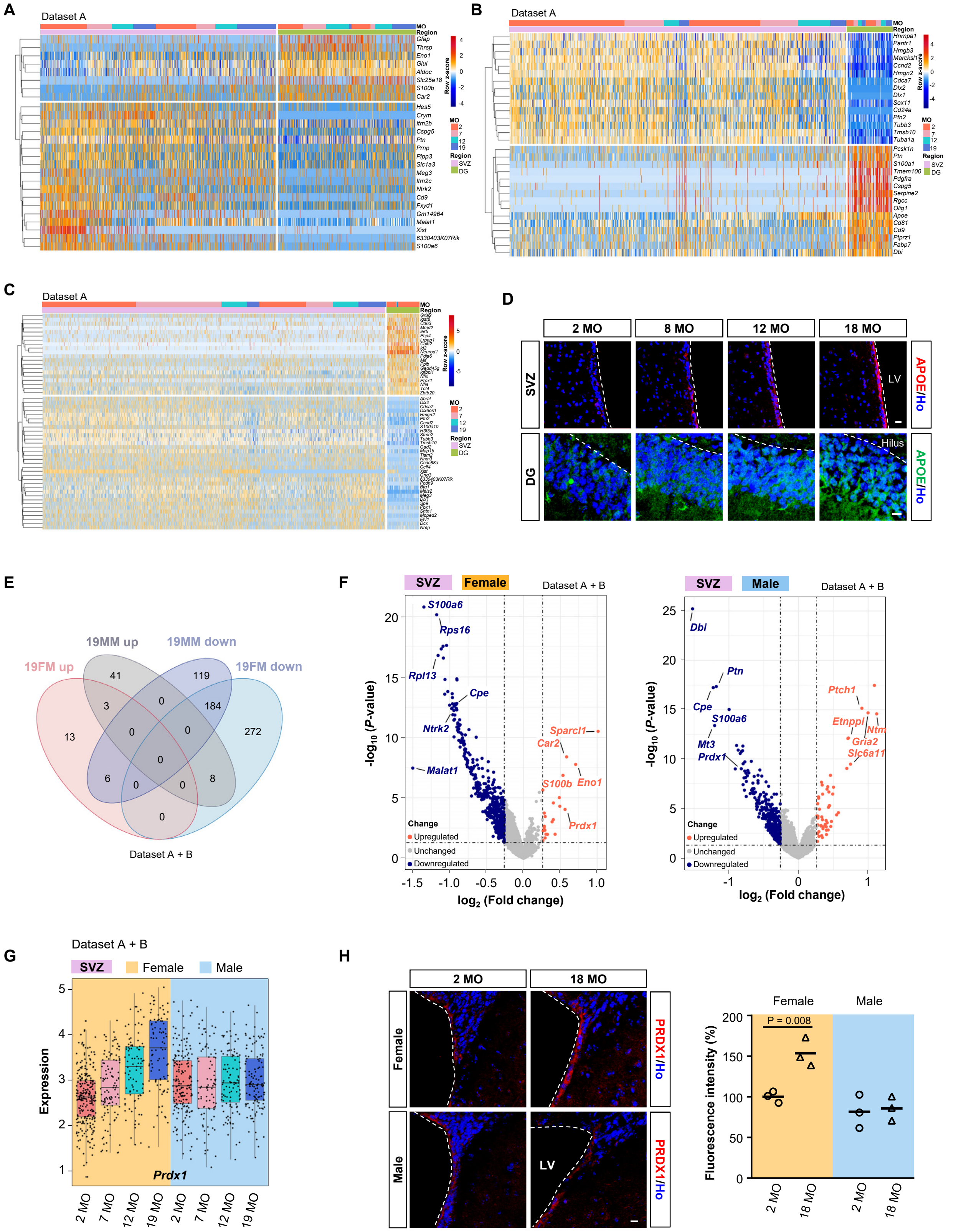

## Supplementary Figure 3

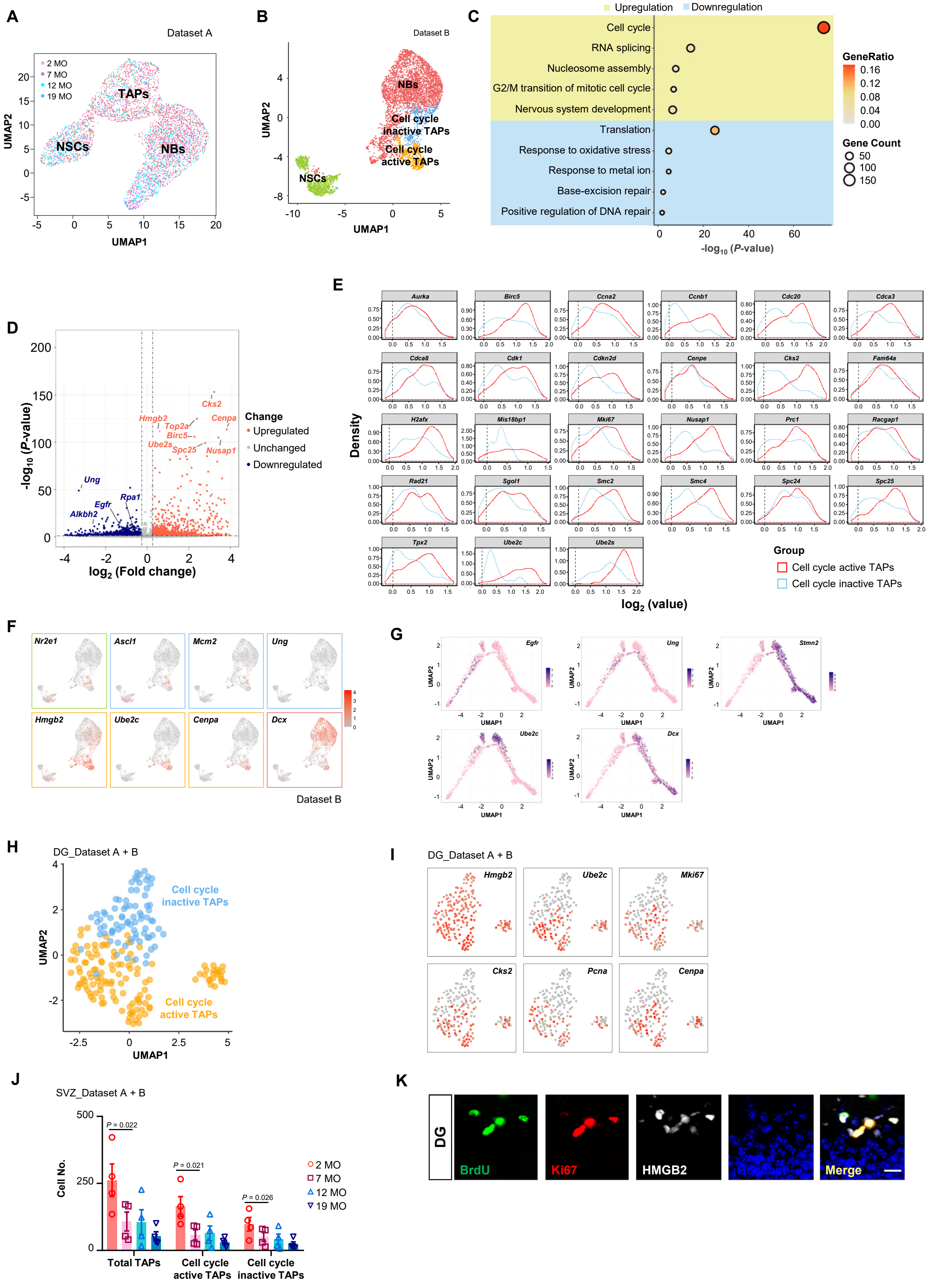

## Supplementary Figure 4

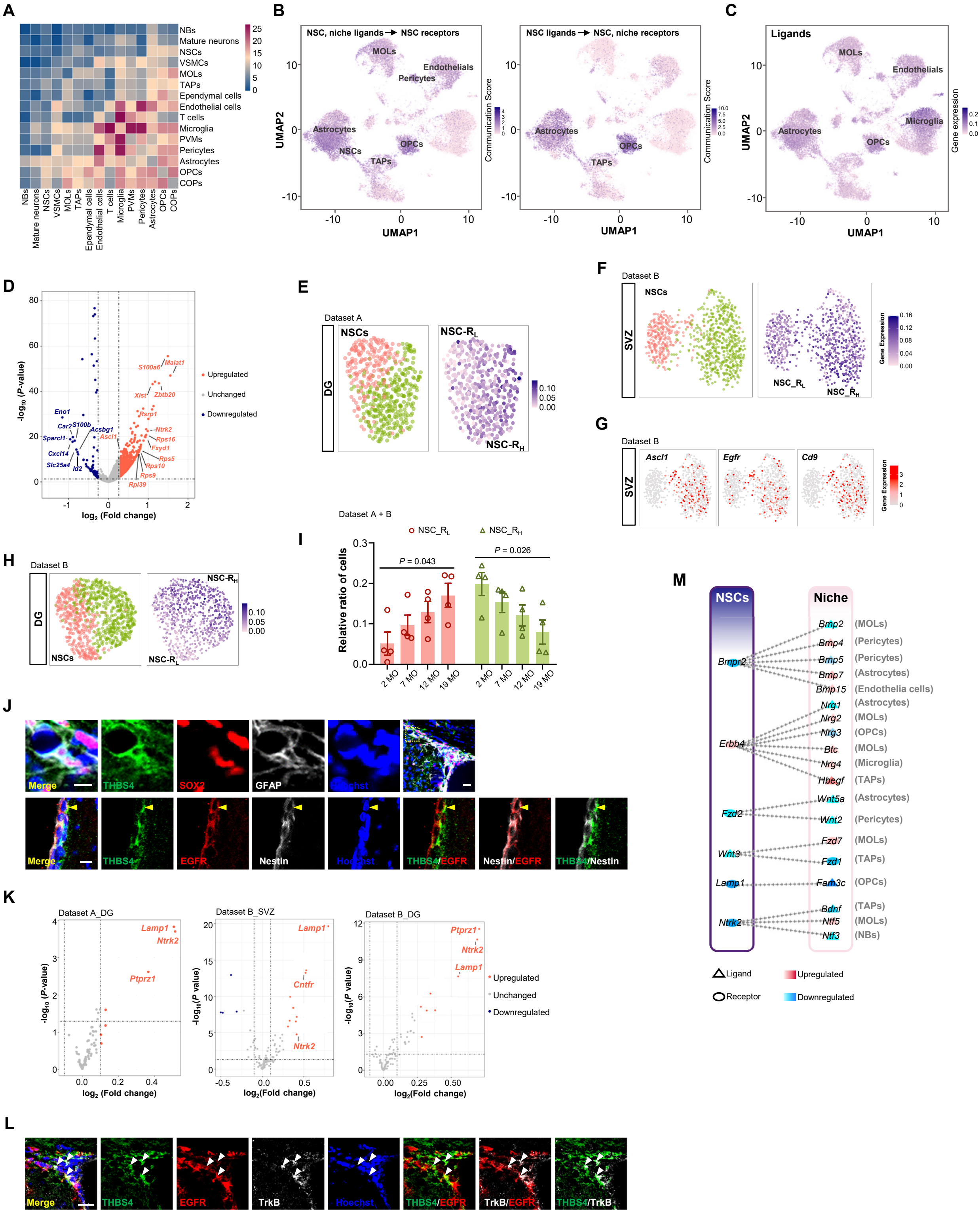

## Supplementary Figure 5

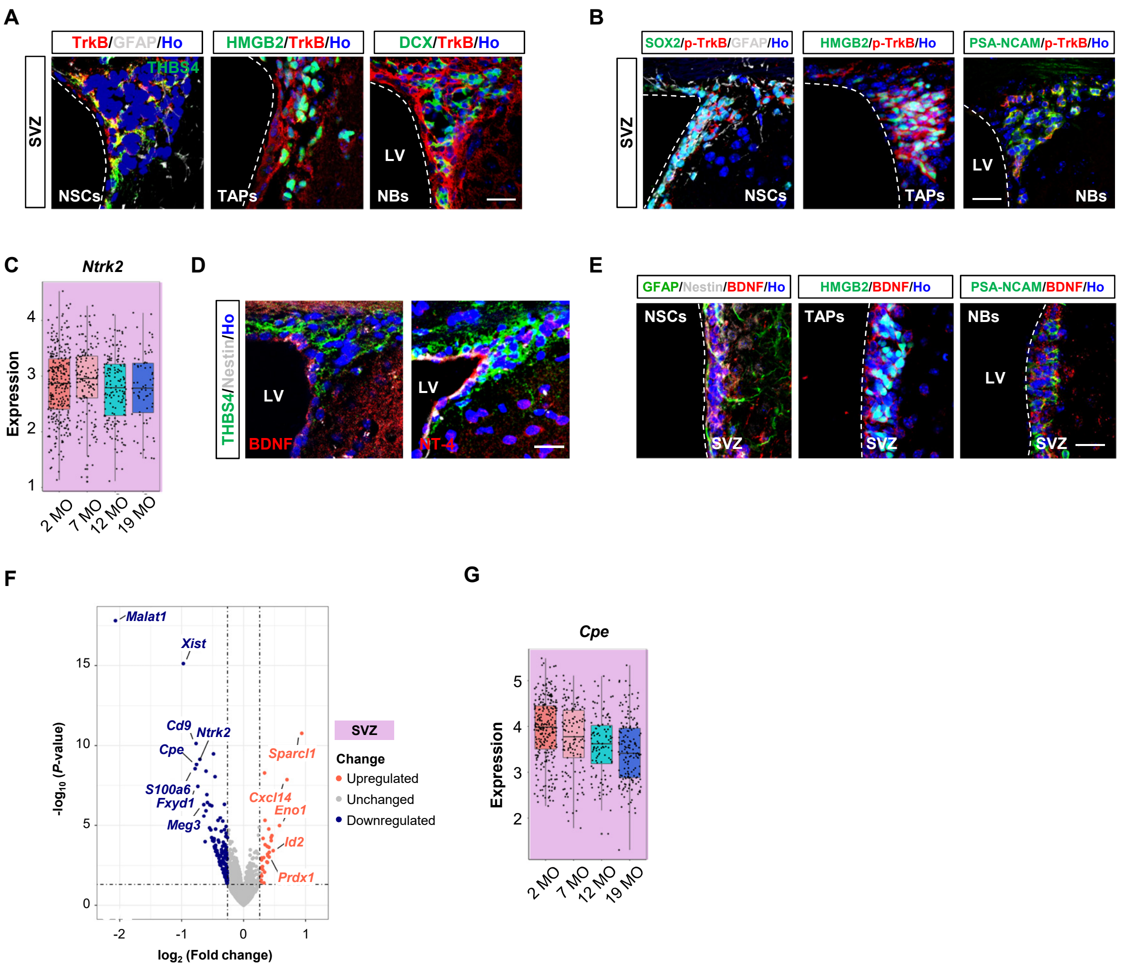

**Supplementary Figure 6**

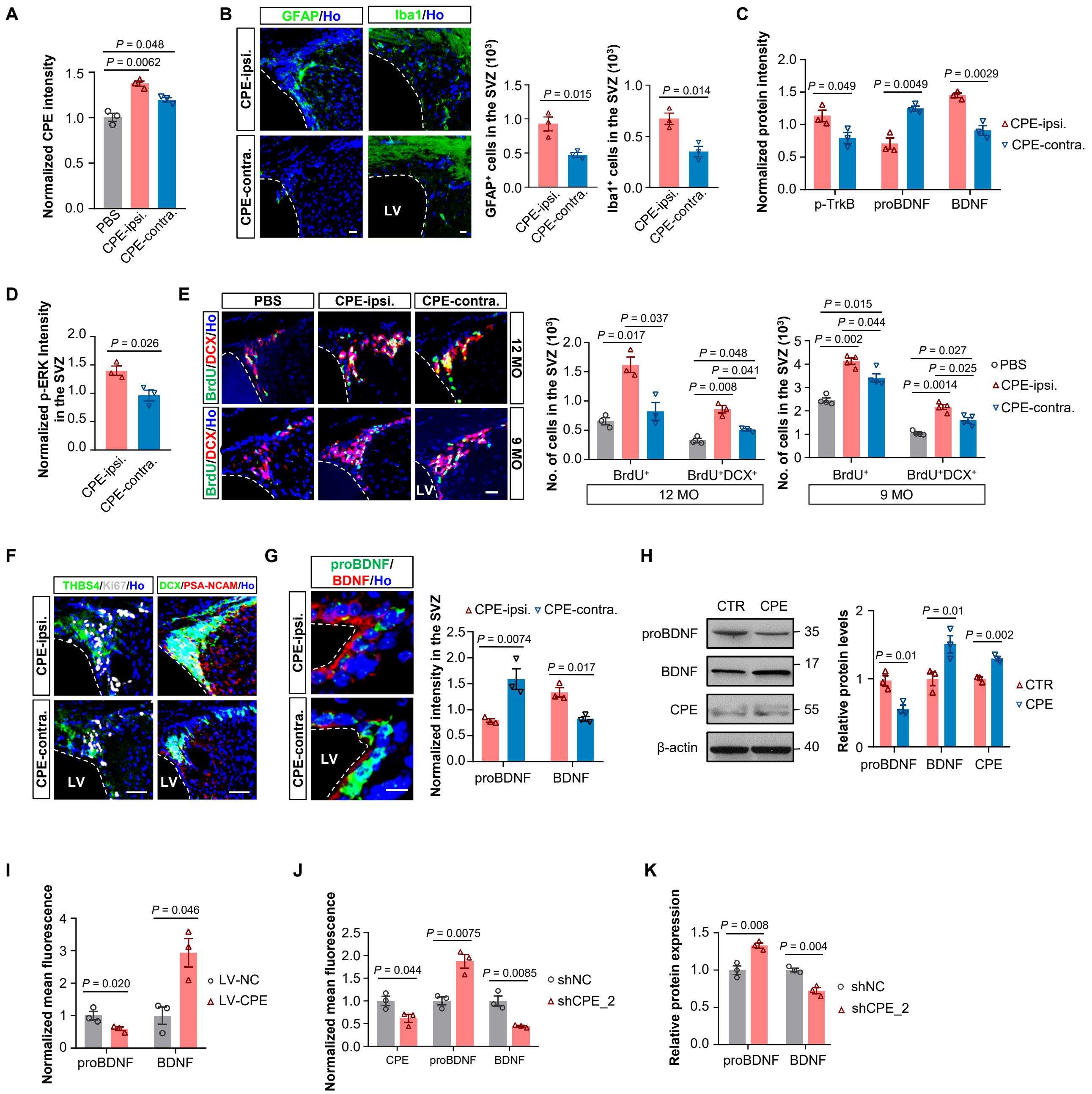

## Supplementary Figure 7

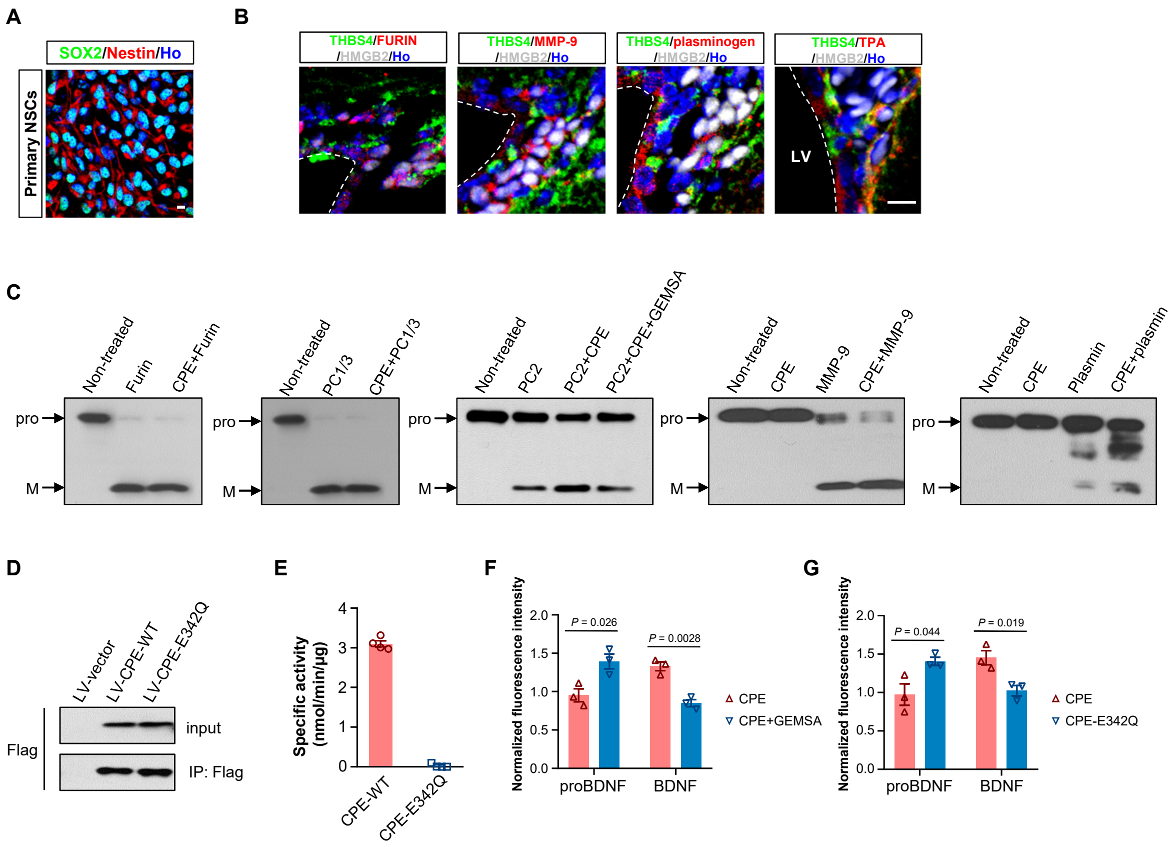

Supplement: lnad015_suppl_Supplementary_Materials [file lnad015_suppl_Supplementary_Materials.pdf]
